# Identification of the *Toxoplasma gondii* mitochondrial ribosome, and characterisation of a protein essential for mitochondrial translation

**DOI:** 10.1111/mmi.14357

**Published:** 2019-07-24

**Authors:** Alice Lacombe, Andrew E. Maclean, Jana Ovciarikova, Julie Tottey, Alexander Mühleip, Paula Fernandes, Lilach Sheiner

**Affiliations:** ^1^ Wellcome Centre for Integrative Parasitology University of Glasgow 120 University Place Glasgow G12 8TA UK; ^2^ UMR 1282 ISP INRA‐Université François Rabelais de Tours Nouzilly France; ^3^ Department of Biochemistry and Biophysics Stockholm University Stockholm Sweden

## Abstract

Apicomplexan parasites cause diseases such as malaria and toxoplasmosis. The apicomplexan mitochondrion shows striking differences from common model organisms, including fundamental processes such as mitochondrial translation. Despite evidence that mitochondrial translation is essential for parasite survival, it is largely understudied. Progress has been restricted by the absence of functional assays to detect apicomplexan mitochondrial translation, a lack of knowledge of proteins involved in the process and the inability to identify and detect mitoribosomes. We report the localization of 12 new mitochondrial proteins, including 6 putative mitoribosomal proteins. We demonstrate the integration of three mitoribosomal proteins in macromolecular complexes, and provide evidence suggesting these are apicomplexan mitoribosomal subunits, detected here for the first time. Finally, a new analytical pipeline detected defects in mitochondrial translation upon depletion of the small subunit protein 35 (*Tg*mS35), while other mitochondrial functions remain unaffected. Our work lays a foundation for the study of apicomplexan mitochondrial translation.

## Graphical abstract

The apicomplexan mitochondrion is divergent and essential yet poorly studied. Mitochondrial translation is predicted to utilise ribosomes assembled from fragmented rRNA but this was never shown. Lack of tools to study mitochondrial translation in apicomplexan has been a long‐standing challenge. Tagging and depletion of *T. gondii* mitoribosomal proteins enabled the first detection of a ribosomal complex, and provided proof of principle for a new mitochondrial translation analytic pipeline.

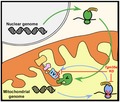

## Introduction

Mitochondria are organelles of central importance to eukaryotic cells, providing key nutrients and metabolites. Mitochondria are present in virtually all eukaryotic cells, excluding a rare case of secondary loss (Karnkowska *et al.*, 2016; Karnkowska and Hampl, 2016). The recent interest in evolutionary cell biology has resulted in an increasing appreciation of the diverse features of mitochondria found in divergent organisms, which includes differences in universal pathways such as protein translation (e.g. Ramrath *et al.*, 2018). However, most of our knowledge of mitochondrial biology is still based on studies in organisms that represent a small proportion of the range of eukaryotic diversity, thus limiting our understanding. One of the challenges to the study of mitochondrial biology in diverse organisms is the lack of tools to characterize and monitor the different mitochondrial functions and biogenesis pathways.

Apicomplexa is a phylum of parasitic protists with high impact on human health globally, which includes the malaria causing *Plasmodium* spp and the causative agent of toxoplasmosis, *Toxoplasma gondii*. Apicomplexans represent one of the lineages whose mitochondrial biology is understudied despite numerous pieces of evidence of its essential role in parasite biology. The apicomplexan mitochondrion hosts an array of essential metabolic pathways (e.g. reviewed here: van Dooren *et al.*, 2006; Sheiner *et al.*, 2013). In addition, its central role in parasite survival is highlighted in recent screens for genes important for fitness in *T. gondii* (Sidik *et al.*, 2016) and *Plasmodium berghei* (Bushell *et al.*, 2017). Both screens showed that high numbers of genes encoding mitochondrial proteins are important for fitness. Finally, mitochondrial pathways are the focus of drug discovery studies (e.g. Srivastava *et al.*, 1997; Nilsen *et al.*, 2013; Phillips *et al.*, 2015; Ke and Mather, 2017), including evidence that mitochondrially encoded proteins are promising targets for the development of antimalarials that may circumvent the emergence of drug resistance (Goodman *et al.*, 2016; Klug *et al.*, 2016). Mitochondrial translation is a prerequisite for the execution of these functions, and it has been suggested as potential target for drugs in itself (e.g. Johnson *et al.*, 2011; Gupta *et al*., 2014). It is, therefore, ever more pressing to expand the studies of mitochondrial translation in these organisms. In this work, we focus on *T. gondii*, a tractable model organism for the phylum.

While mitochondrial translation is considered a fundamental pathway in eukaryotes, it appears to be highly unusual in apicomplexans. For example, *Plasmodium* ribosomal RNAs display a high degree of fragmentation (Feagin *et al.*, 2012; Hillebrand *et al.*, 2018) and it is unclear how these small RNA fragments come together to form a ribosome. Moreover, while evidence for the mitochondrial import of ribosomal and ribosome assembly proteins start to emerge (Ke *et al.*, 2018; Gupta *et al.*, 2018a; 2018b), no evidence that they form a macromolecular‐complex that could correspond to a ribosome has been produced. Nevertheless, it is expected that mitochondrial translation is active in apicomplexans. This was inferred from a combination of findings. The apicomplexan mitochondrion encodes three proteins that are all subunits of complexes of the mitochondrial electron transport chain (mETC): cytochrome *b* subunit (Cytb) of complex III, and cytochrome *c* oxidase subunits I and III (CoxI and III) of complex IV (Suplick *et al.*, 1988; Vaidya *et al.*, 1989; Feagin, 1992; Lin *et al.*, 2011; He *et al.*, 2014; Ogedengbe *et al.*, 2014; Cinar *et al.*, 2015). A study in *P. falciparum* asexual stages showed that an active mETC is essential for replenishing ubiquinone, which is needed to accept electrons from the parasite dihydroorotate dehydrogenase (DHODH) enzyme (Painter *et al.*, 2007). This was shown by an elegant cross species complementation with the yeast DHODH which uses fumarate as the electron acceptor (Painter *et al.*, 2007). In addition, it was found that resistance to atovaquone, which is an antimalarial drug that inhibits the parasites mETC, is acquired through mutations within the mitochondrial gene encoding the Cytb subunit (Siregar *et al.*, 2008). Together, these observations highlight an active mitochondrial translation system essential for the mETC to function and thus for parasite survival. Further support is provided indirectly, by the observed import of tRNAs into the mitochondrion of *T. gondii* and *P. falciparum* (Esseiva *et al.*, 2004; Pino *et al.*, 2010; Sharma and Sharma, 2015). Finally, it was recently shown that a putative mitochondrial ribosomal (mitoribosome) protein is essential for *P. falciparum* growth, and that its depletion results in mitochondrial biogenesis defects (Ke *et al.*, 2018). However, the observed phenotype upon gene depletion could not be rescued via complementation with yeast DHODH (Ke *et al.*, 2018), leaving some uncertainties about the link between mitochondrial translation and mETC function in *Plasmodium* asexual blood stages. No direct evidence for this link, or indeed for active mitochondrial translation, is available in *T. gondii*.

Mitoribosomes are composed of two macromolecular complexes, the large subunit (LSU) and small subunit (SSU). Mitoribosomes further contain ribosomal RNA (rRNA) molecules of varying numbers, with apicomplexan being the highest number and the smallest rRNA fragments known (Feagin *et al.*, 1997). The rRNAs are mostly encoded within the mitochondrial genome, while most mitoribosomal proteins, and in apicomplexans, all of them, are encoded in the nuclear genome. The proportion of proteins and rRNA making up mitoribosomes changes drastically among divergent organisms, and this results in variability of ribosome diameter, kilo Dalton size and number of mitoribosomal proteins (e.g. Ramrath *et al.*, 2018 vs Greber *et al.*, 2015). As expected, those parameters affect the mechanism of mitoribosomal functions, highlighting the importance of experimentally addressing these details in varying systems.

Here, we report the identification of 10 new *T. gondii* mitochondrial proteins found using an *in silico* search designed to identify mitochondrial housekeeping components. These include four mitoribosomal proteins, and we localise two additional mitoribosomal proteins predicted in other studies. We demonstrate that three of those *T. gondii* mitoribosomal proteins are each part of a macromolecular complex, likely one or both mitoribosome subunits, providing the first evidence for the assembly of a mitochondrial ribosome in an apicomplexan parasite, and a first indication of its size. We find that two *T. gondii* mitoribosomal proteins are important for parasite growth, and that shortly after the depletion of the small subunit protein 35 (*Tg*mS35), a mitochondrial complex, whose subunits are encoded in the mitochondrial genome, shows decreased activity, while other complexes remain active. These observations provide evidence that *T. gondii* mitochondrial translation is active and that it depends on *Tg*mS35.

## Results

### Identification and localisation of 10 new *Toxoplasma* mitochondrial proteins

In *T. gondii* tachyzoites, organelles biogenesis is tightly synchronised with the cell cycle and each organelle, including the mitochondrion, has a narrow window of time during cell division when it divides (Nishi *et al.*, 2008). This suggests that proteins needed for mitochondrial growth and expansion may co‐express during the narrow window of mitochondrial growth before division. These proteins would include components of the mitochondrial protein import machinery, allowing nuclear‐encoded proteins to get into the mitochondrion, and mitochondrial translation components, allowing synthesis of the mitochondrial encoded proteins. We define these collectively here as ‘mitochondrial housekeeping proteins’.

We wished to expand the repertoire of known mitochondrial housekeeping proteins, and especially translation components. A challenge is that not all components can be identified via homology searches nor through mitochondrial targeting predictions. It was previously shown that patterns of mRNA co‐expression during the *T. gondii* tachyzoite cell cycle have functional predictive power (Behnke *et al.*, 2010; Sheiner *et al.*, 2011; Huynh and Carruthers, 2016). This was demonstrated for proteins involved in nuclear genome maintenance, microneme adhesins, rhoptry effectors (Behnke *et al.*, 2010), apicoplast biogenesis (Sheiner *et al.*, 2011) and host cell invasion (Huynh and Carruthers, 2016). We reasoned that mRNA expression patterns could be used to generate a list enriched with genes expressing components of mitochondrial housekeeping pathways. At the time of performing this search, the apicomplexan mitochondrial protein import pathway had the most components predicted among the mitochondrial housekeeping pathways, we therefore assembled a group of 14 T*. gondii* homologs of these components based on the prediction made in (van Dooren *et al.*, 2006) (Table S1 – top). We queried the microarray data generated from synchronised *T. gondii* tachyzoites (Behnke *et al.*, 2010) with these 14 baits. Three genes (*TGME49_274090/260850/251780*) did not show cyclical expression pattern during the cell cycle, therefore we proceeded with the remaining 11 (Table S1). We queried the microarray data for mRNAs whose pattern of expression correlates with that of each of these 11 baits using either Euclidian distance or Spearmann correlation (as described in the materials and methods section). This search identified 279 genes (Fig. S1A and Table S2 – Sheet 1 – column A). Our search criteria were such that each bait was used to query for similar but not identical mRNA expression pattern, namely a search with a given bait does not identify itself. Despite this restriction, the overall resulting dataset includes 10 of the 11 baits used for the searches (Tables S1 and S2 – sheet 1 – columns C‐D), meaning that queries with some baits identified the other baits, providing validation for this approach. Moreover, three other genes encoding protein import components that were not used in the bait list (since they were not predicted at the time the search was performed) are also found in this dataset (Tables S1 – bottom and S2 – Sheet 1 – columns C‐D). As a negative control, we examined 5 lists of 279 T*. gondii* genes generated at random (by sorting all the protein encoding genes in ToxoDB by size and selecting lists of 279 geneIDs from across the size range – see Table S2 – Sheet 1 – Columns F‐J). We found that the 13 protein import components, and the 10 new mitochondrial proteins we validated in this study below (Table S3), that are all found in our 279 dataset, are underrepresented in the five random gene lists (none of them found in four lists, and five are found in one list) (Table S2 – Sheet 1 – columns F‐P). These observations provide support for an enrichment of mitochondrial housekeeping proteins in our dataset compared to random datasets of the same size. Moreover, while protein import components and the herein newly identified proteins are enriched, genes encoding mitochondrial proteins that function in other known (metabolic and physiological) pathways that are not expected to co‐express with mitochondrial housekeeping pathways are underrepresented in the resulting dataset (Fig. S1B and Table S2 – sheet 1 – columns R‐AA), providing support to this approach's specificity.

**Figure 1:**
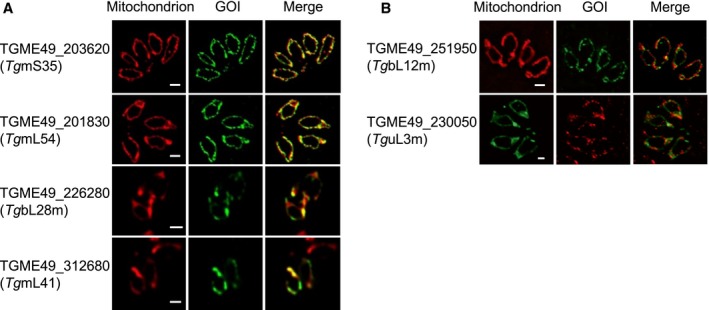
Mitochondrial localisation of putative mitochondrial ribosomal proteins by epitope tagging. A. Localisation through fluorescence microscopy analysis of 4 gene‐products predicted to encode for mitoribosomal proteins identified in our search. B. Localisation of 2 additional gene‐products predicted to be mitochondrial ribosomal proteins not found in our search. For both panels, mitochondria are marked with anti‐TgMys or anti‐TOM40; the mentioned GOI (genes of interest) are marked with anti‐HA, anti‐FLAG or anti‐Strep. Scale bar 1 μm. Images in A also appear in Fig. S2. [Colour figure can be viewed at https://www.wileyonlinelibrary.com]

To sharpen our focus on mitochondrial translation, we added another filter to our search: we postulated that *Cryptosporidium* spp, that are the only known species among the apicomplexans that lost their mitochondrial genome, likely lost their mitochondrial translation pathway that is otherwise conserved among other apicomplexans. We queried the datasets for genes that do not have orthologs in any of the three genomes available for *Cryptosporidium* spp, but that do have orthologues in the genome of *Plasmodium falciparum* 3D7. This search identified 43 genes (Table S2 – sheet 2 – column A). In support of some enrichment of translation components in this dataset, we found 5 of the 35 predicted mitochondrial ribosomal proteins (Gupta *et al.*, 2014) in the 43‐gene dataset, while none of them is found in five lists of 43 randomly chosen genes (Table S2 – sheet 2 – columns C‐O). We attempted to localise the protein product of 14 genes from this focused dataset. We primarily used endogenous gene tagging via single homologous recombination to introduce a triple‐HA epitope tag fusion at the protein c‐terminal end (Huynh and Carruthers, 2009; Sheiner *et al.*, 2011). Where endogenous tagging was unsuccessful, we assessed the localisation via expression of 3xMyc‐tagged cDNA from a heterologous promoter. Among the 12 genes for which we obtained detectable signal (Table S3), 10 were mitochondrial (Fig. S2 and Fig. 1A).

### Detection of the *T. gondii* mitochondrial ribosome

**Figure 2:**
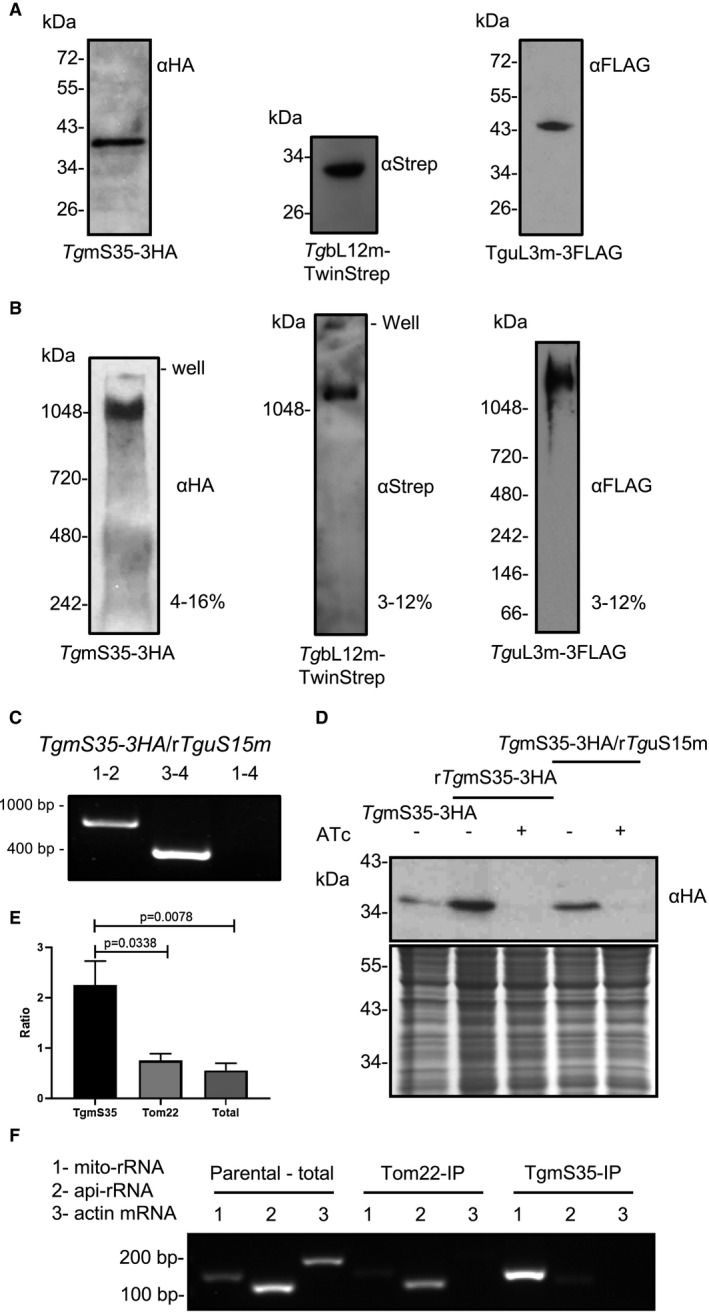
Detection of the *T. gondii* mitochondrial ribosome. A. Protein immunoblot analysis of endogenously tagged TgmS35, TgbL12m and TguL3m from total cell lysate separated by SDS‐PAGE and detected using anti‐HA/Strep/FLAG antibodies. B. Total cell lysate separated by blue‐native PAGE and immunoblotted to detect TgmS35, TgbL12m and TguL3m with anti‐HA/Strep/FLAG antibodies. C. Validation of the promoter integration in the *Tg*mS15 locus via PCR analysis using primers 1, 2, 3, and 4, represented in Fig. S4D. Western blot (top panel) of TgmS35‐3xHA in lines where *Tg*mS35 is under its native promoter (TgmS35‐3HA) or where*Tg*mS35 or *Tg*uS15m are under regulatable promoters (r*Tg*mS35‐3HA and *Tg*mS35‐3HA/r*Tg*uS15m respectively). Low panel shows instant blue staining for loading control. E. comparison of results from RT‐PCR performed with primers for a mitochondrial rRNA sequence (Fig. S5) (mito‐rRNA), for an apicoplast rRNA sequence (api‐rRNA) and for a cytosolic mRNA (actin). Template is RNA extracted from total cell lysate of TATi∆ku80 (total), from IP of TgTom22 (Tom22) or from IP of TgmS35 (TgmS35). F. An example RT‐PCR experiment performed with primers for a mitochondrial rRNA sequence (Fig. S5) (mito‐rRNA), for an apicoplast rRNA sequence (api‐rRNA) and for a cytosolic mRNA (actin). Template is RNA extracted from total cell lysate of TATi∆ku80 (Parental – total), from IP of TgTom22 (Tom22‐IP) or from IP of TgmS35 (TgmS35‐IP).

Among the above 10 genes we localised to the *T. gondii* mitochondrion, 4 are predicted to encode mitoribosomal proteins (Table 1 and Fig. 1A): TGME49_201830/226280/312680 are predicted to encode the mitochondrial large subunit components 54, 28 and 41, respectively, thus we named them *Tg*mL54, *Tg*bL28m and *Tg*mL41; TGME49_203620, while annotated as hypothetical protein in http://ToxoDB.org, is a putative homolog of the mitochondrial small subunit 35 (mS35) (Gupta *et al.*, 2014; Greber and Ban, 2016), we, therefore, named it, *Tg*m*S35*. To provide support for this prediction, we tested the gel migration patterns of endogenously triple‐HA tagged *Tg*m*S35*. While *Tg*m*S35*'s protein product migrates around 38 KDa under denaturing conditions (Fig. 2A), around the predicted size of the part of the protein that is conserved among apicomplexans (Fig. S3), it migrates with a high molecular weight (>1000 KDa) complex, when separated under native conditions using blue‐native PAGE (Fig. 2B). The migration under native conditions indicates a size that could well correspond to the mitochondrial ribosomal complex.

**Table 1:** Putative mitoribosomal proteins investigated in this study.

Name	Gene ID	SSU/LSU	Source	Tag	Mitoprot	Predotar	CRISPR score	Proximity tagging proteome
*Tg*mS35	TGME49_203620	SSU	Expression screen; Gupta *et al.* (2014)	HA	0.9734	0.53	−4.66	No*
*Tg*mL54	TGME49_201830	LSU	Expression screen	HA	0.9991	0.43	−4.94	No
*Tg*bL28m	TGME49_226280	LSU	Expression screen; Gupta *et al.* (2014)	myc	0.9971	0.65	−4.91	Yes
*Tg*mL41	TGME49_312680	LSU	Expression screen	myc	0.9388	0.14	−1.34	No
*Tg*bL12m	TGME49_251950	LSU	Gupta *et al.* (2014)	Strep	0.9672	0.08	−3.60	Yes
*Tg*uL3m	TGME49_230050	LSU	Gupta *et al.* (2014)	FLAG	0.8966	0.02	−4.50	Yes
*Tg*uS15m	TGME49_216040	SSU	Gupta *et al.* (2014)	N/A	0.9885	0.83	−4.51	Yes

Putative mitoribosomal proteins were identified through our mRNA expression based screen (Fig. S1) and/or identified bioinformatically in Gupta *et al.* (2014) ‘Reduced ribosomes of the apicoplast and mitochondrion of *Plasmodium* spp. and predicted interactions with antibiotics’. The putative mitoribosomal proteins were named according to the new nomenclature for ribosomal proteins (Ban *et al*., 2014; Greber and Ban, 2016). Mitochondrial targeting predictions were done using MitoProt II (https://ihg.gsf.de/ihg/mitoprot.html) and Predotar (https://urgi.versailles.inra.fr/predotar/). The presence (Yes) or absence (No) of the gene product in the proximity tagging‐based mitochondrial proteome is shown (Seidi *et al.*, 2018) (No – no mass spectrometry data; No* – mass spectrometry data found but the gene is not included in the final list of 421 genes). The fitness score is based on the whole genome CRISPR/CAS9 screen (Sidik *et al.*, 2016).

We further tagged additional predicted ribosomal proteins from (Gupta *et al.*, 2014) via endogenous tagging: TgbL12m (TGME49_251950) was tagged with TwinStrep, and TguL3m (TGME49_230050) was tagged with triple‐FLAG (Table 1); and confirmed their mitochondrial localizations (Fig. 1B). Like TgmS35, under denaturing conditions these proteins migrate at the expected size of their mature form (Fig. 2A). Upon separation under native conditions all three tagged proteins migrated similarly to one another (Fig. 2B) providing support that all three proteins belong to the same complex, likely the mitoribosome, or that the SSU and LSU complexes are of similar sizes. Moreover, we generated a conditional knock‐down line by replacing the native promoter of the gene encoding the putative *Tg*uS15m (TGME49_216040) (Gupta *et al.*, 2014) (Table 1) with an anhydrotetracycline (ATc) repressible promoter. This manipulation was performed as previously described (Sheiner *et al.*, 2011), however with the modification whereby CRISPR/Cas9 was utilized to enhance integration of the promoter cassette (Fig. S4). The promoter replacement was performed in a parental line where *TgmS35* is endogenously tagged with triple‐HA (*TgmS35‐3HA/rTguS15m*) and was confirmed using PCR (Fig. 2C). Western blot analysis showed that downregulation of *TguS15m* results in depletion of TgmS35 (Fig. 2D), supporting these proteins being part of the same complex.

Finally, we performed immunoprecipitation (IP) experiments with the triple‐HA‐tagged TgmS35 and used the IP fraction to extract RNA, which was then used as template for RT‐PCR. We reasoned that the IP will enrich mitoribosome complexes and, therefore, that mitochondrial rRNA will be enriched in the IP fraction compared to apicoplast rRNA (api‐rRNA, using primers from Biddau *et al.*, 2018) or to a cytosolic mRNA (actin, using primers from Melatti *et al.*, 2019). The RT‐PCR for mitochondrial‐encoded rRNA was performed using primers designed to amplify a sequence that is conserved between a coccidian and an haemosporidian whose mitochondrial DNA genome sequences are published (GenBank accessions: M76611.1 and AB564272.1) (Fig. S5) (mito‐rRNA). As negative controls, we tested RNA extracted from fractions from IP experiments performed with a line where the protein import component TgTom22 is tagged with HA (van Dooren *et al.*, 2016), and RNA extracted from total lysate of the parental line TATi∆ku80. In support of the IP enrichments of organellar rRNA, the PCR for actin did not amplify products from most of the IP fractions (we could observe a band in one of four Tom22‐IP experiments, and in none of seven TgmS35‐IP experiments) while consistently amplifying from total RNA (five times of five experiments). We found that the mito‐rRNA PCR product was consistently more abundant than api‐rRNA (average mito/api ratio 2.85 ± 0.5, *n* = 7) in TgmS35 IP‐fractions compared to the controls (0.75 ± 0.13, *n* = 4, from TgTom22‐IP and 0.56 ± 0.17, *n* = 5, from total lysate) (Fig. 2E shows a graph summarising data from all the experiments and Fig. 2F shows an example of one experiment). These findings provide additional support for *TgmS35* being a part of a ribosomal complex.

Taken together, the size of the complexes marked by tagged TgmS35, TgbL12m and TguL3m, the co‐depletion of TgmS35 upon *Tg*uS15m depletion and the specific enrichment of mitochondrial rRNA in the TgmS35 pull‐down, support the identity of the observed complex to be one of the *T. gondii* mitochondrial ribosome subunits or the full mitoribosome.

### Transient expression of CAS9 results in mitochondrial morphology defects

**Figure 3:**
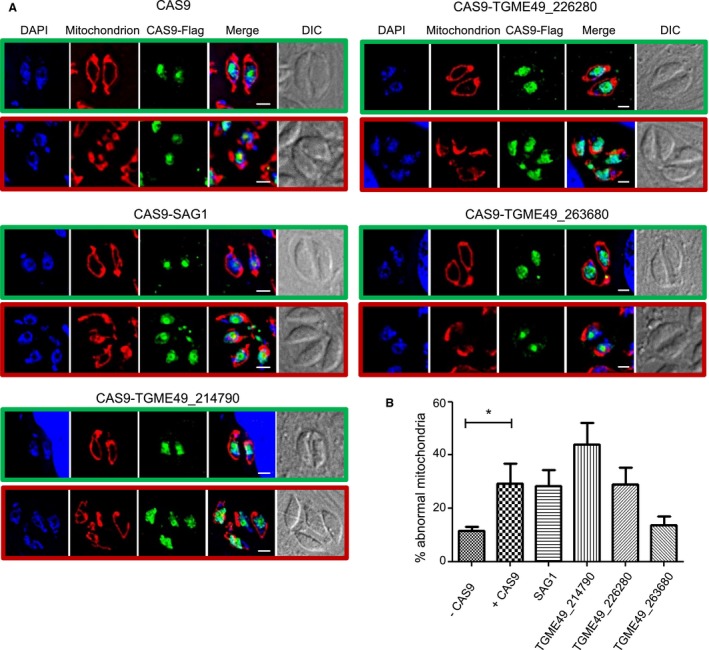
Transient expression of Cas9 results in mitochondria morphology defects. A. Representative immunofluorescence micrographs of parasites transiently expressing CAS9 and sgRNA for the mentioned GOI. Mitochondria in red are marked by anti‐TgMys. CAS9‐FLAG in green is marked with anti‐FLAG. Merge shows DAPI, FLAG and TgMys. Green boxes highlight parasites with wild‐type looking mitochondria. Red boxes highlight parasites with mitochondria that appear to have a morphological defect herein named ‘abnormal mitochondria.’ B. Quantification of vacuole with abnormal mitochondria morphologies for each condition. Bars represent the mean ± SEM (*n* = 3), *p < 0.05 (1‐way ANOVA). [Colour figure can be viewed at https://www.wileyonlinelibrary.com]

Having a group of 12 newly validated mitochondrial proteins to study, we wanted to examine whether transiently expressed CRISPR/Cas9 strategy could be used to provide a rapid read‐out assay to detect protein's involvement in mitochondrial biogenesis. We hypothesised that abnormal changes in mitochondrial morphology upon gene disruption may be an indicator for a defect in mitochondrial biogenesis. We picked three of the new mitochondrial proteins at random (encoded by *TGME49_263680/214790* and *226280*). For each gene, we co‐expressed FLAG‐tagged Cas9 (Sidik *et al.*, 2014) along with a targeting single guide RNA (sgRNA) (Table S4). As control, we expressed the Cas9‐FLAG alone or with sgRNA for the non‐essential SAG1 (*TGME49_233460*) gene for which we do not expect any mitochondrial phenotype upon gene disruption (SAG1sgRNA). At 48 h post co‐transfection, mitochondria were imaged using immunofluorescence and morphologies were analysed (Fig. 3A). We observed elevated mitochondrial abnormalities in all conditions tested except the untreated parasites. While a trend of higher abundance of mitochondrial abnormalities was observed for *TGME49_214790*, the data showed no significant difference from the Cas9‐only and Cas9 + SAG1sgRNA controls (Fig. 3A and B). These data suggest that transient expression of Cas9 results in mitochondrial morphological abnormalities, excluding this strategy as a reliable tool for rapid identification of genes involved in mitochondrial biogenesis.

### The ribosomal proteins *Tg*mS35 and *Tg*uS15m are crucial for parasite fitness and mitochondrial biogenesis

**Figure 4:**
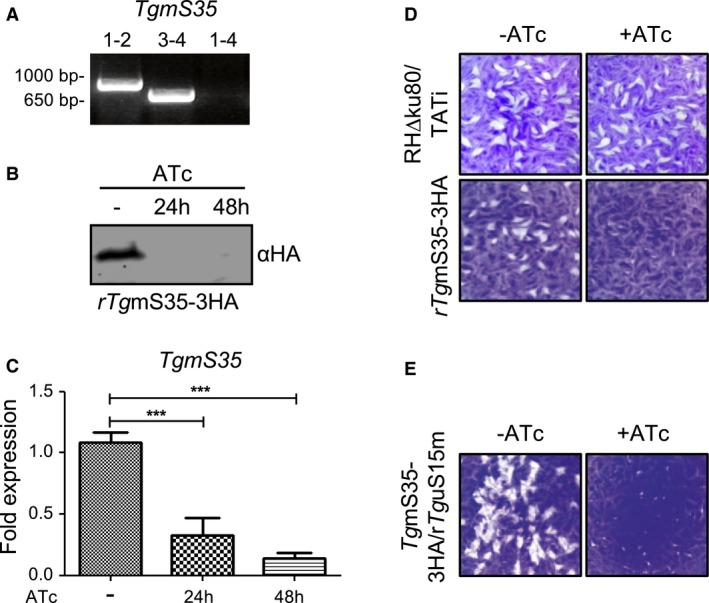
Knock‐down and analysis of *TgmS35*. A. Validation of the promoter integration in the *Tg*mS35 locus via PCR analysis using primers 1, 2, 3, and 4, shown in Fig. S4. B. Protein immunoblot analysis of whole cell lysate from r*Tg*mS35‐3HA after growth in absence (−) or presence of ATc for 24 and 48 h. C. Transcript levels of *Tg*mS35, analysed by qRT‐PCR, in absence (−) or presence of ATc after 24 and 48 h. Bars represent the mean ± SEM (*n* = 5). ***p < 0.0001 D. Plaque assays performed with parental line (RH∆ku80/TATi) or r*Tg*mS35‐3HA parasites grown for 9 days in the absence (−) or presence (+) of ATc. E. Plaque assays performed with r*Tg*mS15 parasites grown for 9 days in the absence (−) or presence (+) of ATc. [Colour figure can be viewed at https://www.wileyonlinelibrary.com]

Due to the complication encountered in analysing mitochondrial biogenesis defects in parasites transiently expressing Cas9, we proceeded with functional analysis using stable genetic manipulation. We generated a *Tg*m*S35* conditional knock down line by replacing its native promoter with an ATc repressible promoter as above (Fig. S4) in the line where *Tg*m*S35* is endogenously triple‐HA‐tagged. We named this line *rTgmS35‐3HA*. Inducible promoter integration was confirmed by PCR (Fig. 4A) and ATc‐induced down‐regulation was confirmed by qPCR and by western blot using the triple‐HA tag (Fig. 4B and C). We monitored parasite growth by plaque assay which revealed a severe growth defect in response to *Tg*m*S35* depletion; however, small plaques revealed that parasites can still grow slowly (Fig. 4D). The same growth phenotype was observed upon depletion of *TguS15m* (Fig. 4E), suggesting this may be a typical mitoribosome depletion phenotype.

**Figure 5:**
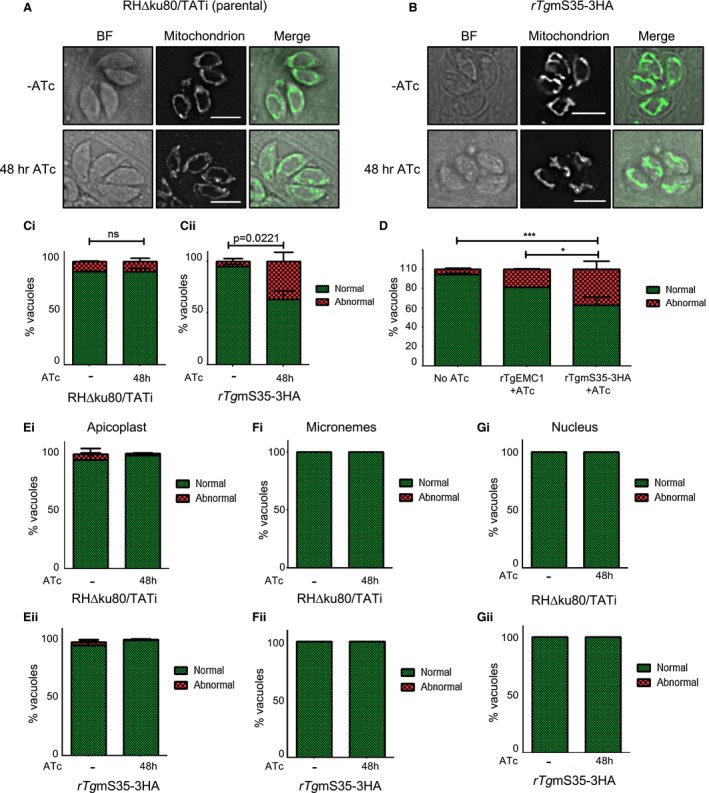
Morphological analysis of organelles under TgmS35 depletion. A/B. Immunofluorescence micrographs taken with the RH∆ku80/TATi parental/rTgmS35‐3HA line, respectively, grown in the presence or absence (−) of ATc for 48 h, showing examples for untreated (‐ATc) or treated (48 h ATc) mitochondrial morphologies. C. Quantification of parasite vacuoles with ‘normal’ (green) vs ‘abnormal’ (red) mitochondrial morphologies after growth of the RH∆ku80/TATi parental (Ci) or the rTgmS35‐3HA (Cii) lines without (−) or with ATc for 48 h. Graphs represent mean ± SEM for *n* = 3 independent experiments; analysed by t test. D. Quantification of ‘normal’ vs ‘abnormal’ mitochondria containing vacuoles after growth of rTgmS35‐HA or rTgEMC1 without (−) or with ATc for 48 h. Graph represents mean ± SEM for *n* = 3 independent experiments for rTgEMC1 + ATc and rTgmS35‐3HA + ATc and *n* = 6 for No ATc where data from rTgEMC1 no ATc and rTgmS35‐3HA no ATc were combined; Analysed by ANOVA followed by Tukey's Multiple Comparison Test, *p < 0.05 and ***p < 0.001. (E) Quantification of ‘normal’ and ‘abnormal’ apicoplast morphology in RH∆ku80/TATi parental (i) and rTgmS35‐3HA (ii) grown without (−) or with ATc for 48 h. Graphs represent mean  ± SEM for *n* = 3 independent experiments. Analysed by t‐test (F) Quantification of ‘normal’ and ‘abnormal’ microneme morphology in RH∆ku80/TATi parental (i) and rTgmS35‐3HA (ii) grown without (−) or with ATc for 48 h. (G) Quantification of ‘normal’ and ‘abnormal’ nucleus morphology in RH∆ku80/TATi parental (i) and rTgmS35‐3HA (ii) grown without (−) or with ATc for 48 h. [Colour figure can be viewed at https://www.wileyonlinelibrary.com]

Immunofluorescence analysis detected mitochondrial morphological abnormalities upon *Tg*m*S35* depletion, and this defect was not detected upon ATc treatment of the parental line (Fig. 5A‐C). Moreover, conditional depletion of a component of an unrelated pathway, a putative ER‐mitochondria complex member, EMC1 (TGME49_205740) ((Wideman and Muñoz‐Gómez, 2016), and Ovciarikova and Sheiner unpublished), while deleterious to the parasites (Fig. S6A and B), showed a milder effect on mitochondrial morphology than what we observed upon depletion of TgmS35 (Figs 5D and S6C). Finally, no significant defect was observed for other organelles upon *TgmS35* depletion (we analysed apicoplast, microneme and nucleus, Fig. 5E‐G). Together, these results are consistent with *Tg*m*S35* being important for parasite fitness and mitochondrial biogenesis.

### A new protocol for evaluation of mitochondrial translation shows that *TgmS35* is essential for this pathway

**Figure 6:**
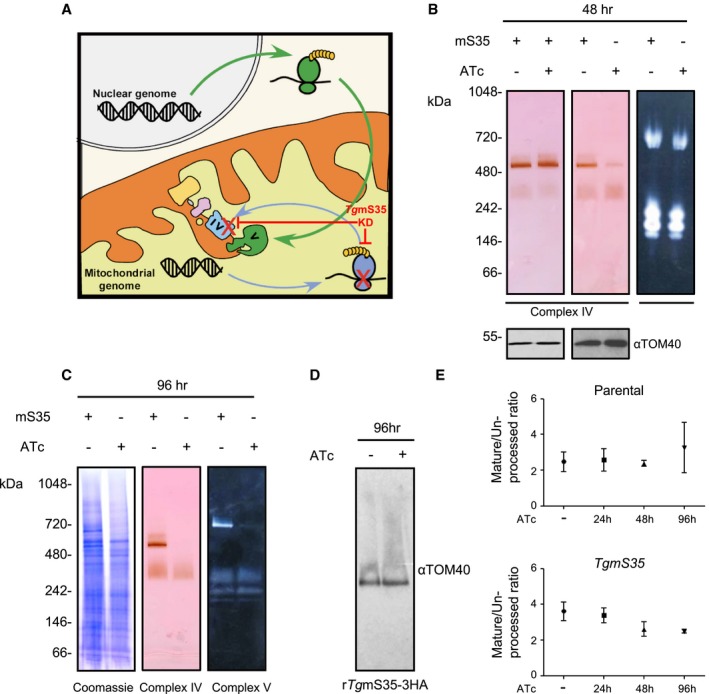
Downregulation of *Tg*mS35 leads to a defect in respiratory complex IV. A. A scheme describing the rational of our assay. Nuclear (grey) encoded proteins translated by the cytosolic ribosome (green) compose complex V, while mitochondrial (yellow) encoded proteins translated by the mitochondrial ribosome (blue) are necessary to assemble complex IV. Depletion of TgmS35 (red) results in reduced activity of complex IV but not V. B. Whole cell (left and middle panel) and enriched mitochondria (right panel) from *Tg*mS35‐3HA (left panel) and from r*Tg*mS35‐3HA (middle and right panel), grown in the absence (−) or presence (+) of ATc for 48 h, separated by high resolution clear‐ native PAGE. Complex IV activity was visualised with cytochrome *c*: DAB staining. Complex V activity was assayed by visualising the precipitation of lead by inorganic phosphate during ATP hydrolysis. Protein immunoblot analysis using anti‐TOM40 of whole‐cell and enriched mitochondria sample was performed as a loading control (bottom panels). C. Mitochondrial enriched fraction from r*Tg*mS35‐3HA grown in the absence (−) or presence (+) of ATc for 96 h, separated by high resolution clear‐native PAGE and stained with Coomassie or for complex IV or complex V activity as above. D. Immunoblot with anti‐TOM40 antibody of mitochondrial enriched fraction from r*Tg*mS35‐3HA grown in the absence (−) or presence (+) of ATc for 96 h, separated by blue‐native PAGE. E. Quantification of the ratio of bands of un‐processed and mature version of Hsp60L‐mDHFR‐cMyc measured in an import assay in parental (top graph) and r*Tg*mS35‐3HA (bottom graph) parasites lines in the absence (−) or presence of ATc for 24, 48 and 96 h. Bars represent the mean ± SEM (*n* = 3 for the parental line, and *n* = 5 for r*Tg*mS35‐3HA). [Colour figure can be viewed at https://www.wileyonlinelibrary.com]

In yeast, mS35 is found at the critical point of mRNA entry into the ribosome (Desai *et al.*, 2017). We hypothesised that depletion of *Tg*m*S35* should result in a mitochondrial translation defect; however, the field lacks an assay to directly monitor mitochondrial translation in *T. gondii*. We, therefore, monitored the assembly and activity of the respiratory chain complex IV, which is directly dependent on the mitochondrial translation of CoxI and III, and compared it to the assembly and activity of complex V, which is not dependent on mitochondrial translation of its subunits (Fig. 6A). Using an enriched mitochondrial fraction, obtained from parasites lysed through nitrogen cavitations, we performed high‐resolution clear‐native PAGE followed by an in‐gel complex IV and V enzymatic assays (Fig. 6B). The signal obtained by complex IV activity assay corresponds to the size reported for *T. gondii* complex IV recently (Seidi *et al.*, 2018) supporting the assay's specificity. To further confirm its identity as complex IV, we performed mass spectrometry analysis of this band which confirmed the complex identity, through confirming the presence of all complex IV components (Table S5). Complex V assay was reported for *T. gondii* previously (Salunke *et al.*, 2018) and we followed the same protocol here. To provide additional confirmation to the complex identity we used an antibody raised against a consensus sequence found in plant, algal and bacterial ATP‐beta subunit of complex V, and which was shown to cross‐react with ATP‐beta from a variety of organisms including *T. gondii* (Huet *et al.*, 2018; Seidi *et al.*, 2018). We first demonstrated the cross‐reactivity of this antibody with *T. gondii* ATP‐beta and showed that lysate from parasites filtered out of their host cells does not contain human ATP‐beta in it (Fig. S7A), and then demonstrated that the bands obtained in the activity assay and with the antibody migrate similarly under native conditions (Fig. S6B).

We performed the two assays at different time points after *Tg*m*S35* depletion (Fig. 6B). We found a decrease in complex IV activity at 48 h and loss of the activity at 96 h after *Tg*m*S35* depletion. In contrast, the activity of complex V is unchanged at 48 h and is only decreased at the 96‐h time point indicating an indirect defect (Fig. 6B). Unlike complex IV, western blot analysis of a native gel from the 96 h time point showed that the protein import complex, translocase of the outer membrane (TOM), migrates at the same size as the untreated line (Fig. 6C). This data suggests that TOM complex, whose components are not encoded in the mitochondrial genome, continue to assemble. To provide further support for TOM complex assembly, we measured protein import into the mitochondrion. A Myc‐tagged DHFR fused to the mitochondrial targeting signal of HSP60 (Hsp60_L_‐mDHFR‐cMyc (van Dooren *et al.*, 2016)) was transiently transfected into the parasites at different time points after *Tg*m*S35* depletion. Upon entry into the mitochondrion, the mitochondrial targeting signal is cleaved off, generating a smaller sized protein which can be visualised by western blot. Upon an import defect, cleavage no longer takes place (van Dooren *et al.*, 2016). We calculated the ratio of signal intensities between the cytosolic un‐cleaved protein and the mitochondrial cleaved protein in each condition. We did not observe any significant protein import defect upon *Tg*m*S35* depletion, including at the 96 h time point (Fig. 6D). Taken together, these observations suggest that *Tg*m*S35* depletion results in a defect in the assembly and activity specifically of a mitochondrial respiratory complex whose subunits are thought to be encoded in the mitochondrial genome, while other complexes continue to assemble correctly and other mitochondrial biogenesis pathways continue to function.

## Discussion

Research in recent years has expanded our understanding of mitochondrial diversity across the eukaryotes. However, there is still a reliance on a handful of models, such as yeast and mammals or such as plants, when studying basic mitochondrial functions. Focusing on these canonical mitochondria gives a skewed view of mitochondrial function and composition. One of the reasons for this focus is the lack of well‐developed, tractable, model organisms outside these key groups. Despite key advances in recent years in studying microbial eukaryotes, there is still a lack of tools for studying mitochondrial biology in non‐model groups. Apicomplexa, which contain the medically important *Plasmodium* and *Toxoplasma* parasites, is such a group where the mitochondria are understudied. The herein described work provides crucial advances to the understanding of mitochondrial translation in this phylum by characterising the mitochondrial ribosome and by establishing an analytical pipeline to identify mitochondrial translation defects.

### Identification of new mitochondrial proteins using an *in silico* search

To fully understand and explore mitochondrial functions it is necessary to create an inventory of experimentally validated mitochondrial proteins. To date only 88 *Toxoplasma* mitochondrial proteins have been experimentally validated (summarised in (Mallo *et al.*, 2018)). Establishing the proteome through organelle isolation has been a challenge to the apicomplexan field partially due to the association between the mitochondrion and the apicoplast (Kobayashi *et al.*, 2007; Nishi *et al.*, 2008; and our unpublished data). While methods that may resolve this complication are being explored (Hata *et al.*, 2018), other strategies that bypass the reliance on organelle isolation are being utilised. Recent biotin‐proximity tagging approaches have made a big step forward by identifying a large proportion of the mitochondrial matrix proteins (Seidi *et al.*, 2018). The number of 421 proposed mitochondrial proteins identified is predicted to be markedly smaller than the total number of mitochondrial proteins estimated through comparison to other species (Panigrahi *et al.*, 2009; Salvato *et al.*, 2014; Smith and Robinson, 2018). Many of these potentially missing proteins are expected to target other mitochondrial sub‐compartments and thus likely do not possess a canonical mitochondrial targeting signal and cannot be predicted using mitochondrial localisation prediction tools. Here, we used an *in silico* search resulting in the identification and validation of 10 new *T. gondii* mitochondrial proteins. These data expand the group of validated mitochondrial proteins by 11%. Only 4 of these 10 are predicted in the Seidi *et al*. (2018) dataset of 421 genes (Table S3), and either 2 or 3 of them are not predicted to target the mitochondrion in two different algorithms (Table S3). In doing so, our search strategy makes a contribution to the ongoing efforts in the field.

Our study focused on mRNAs that co‐express with homologs of the mitochondrial protein import machinery components, some of which were recently validated experimentally (van Dooren *et al.*, 2016). In the same study the authors identified new components of these import complexes that were not included in our bait list (van Dooren *et al.*, 2016). The presence of 3 of these 5 new import components in our 279‐gene dataset (Table S1 bottom) lends support to the power of this strategy to identify mitochondrial protein import components, especially considering none of them are found in five lists of randomly chosen 279 genes (Table S2 – Sheet 1 – columns F–P). This observation raises the possibility that some other genes in the dataset may take part in protein import. A previous study showed that at least one of the mitochondrial protein translocation complexes has comparable size to other organisms (van Dooren *et al.*, 2016). While homologs of many components known in other organisms are missing in *T. gondii* (van Dooren *et al.*, 2016), suggesting that currently unknown components are part of this machinery and that they may be parasite specific. The 109 apicomplexan unique genes in our dataset (Table S6, column C) provide candidates for those missing pieces. Other characteristics of components of the mitochondrial protein import machinery is absence of a canonical mitochondrial targeting signal, and presence of transmembrane domains. Table S6 summarises the prediction of these features in the 279 genes. Another pathway whose components may co‐express with protein import components is mitochondrial tRNA import (Esseiva *et al.*, 2004; Pino *et al.*, 2010; Sharma and Sharma, 2015), as in some organisms these two pathways are linked (Tschopp *et al.*, 2011; Seidman *et al.*, 2012). Mitochondrial tRNA import is likely essential for mitochondrial translation, for which components are enriched in the 43‐gene dataset, proposing these 43 genes as candidate for component of this pathway.

### Mitochondrial morphology captured by immunofluorescence is linked to mitochondrial functional defects in *Toxoplasma*

In model organisms, mitochondrial morphology and function are tightly linked. In support of this also being the case in *Toxoplasma,* recent studies reported rapid mitochondrial remodelling in respond to drastic changes in the growth environments (Charvat and Arrizabalaga, 2016; Ovciarikova *et al.*, 2017). The morphological defect observed here upon *Tg*mS35 depletion provides additional support (Fig. 5). By analysing another line where an essential ER protein is depleted, we also demonstrate that this effect is specific to defect in mitochondrial function rather than a general outcome of parasite death (Figs 5D and S6C). For this reason, we hoped to use mitochondrial morphological changes as a preliminary indicator for mitochondrial functional defects. However, we found that transient expression of the Cas9 endonuclease in *T. gondii* induces mitochondrial morphology defects (Fig. 3). This observation should be considered when analysing mitochondrial phenotypes under these conditions.

### Observation of the mitochondrial ribosome and characterisation of a new ribosomal component demonstrate the crucial role of mitochondrial translation in *T. gondii* survival

A recent structure of the mitochondrial ribosome of *Trypanosoma brucei* showed an evolutionary shift towards a higher ribosome protein content rather than the RNA heavy complexes of mammalian ribosomes (Ramrath *et al.*, 2018). These findings from *T. brucei* illustrates the importance of studying basic cellular components in a diverse array of models, however, this is not possible without detecting mitochondrial ribosomes and validating mitochondrial ribosomal proteins. Among the 10 genes we localised here (Fig. S2), 4 were predicted to be ribosomal subunits (Table 1), we localised 2 other predicted mitoribosomal proteins (Fig. 1 and Table 1) and validation of mitochondrial localisation was provided for 4 more components in the proximity tagging study (Seidi *et al.*, 2018). Table S7 summarises the localisation information available for predicted mitoribosomal proteins in *T. gondii* as well as provide new notations in agreement with the fields' standard (Greber and Ban, 2016).

Blue‐native separation of endogenously tagged *TgmS35*, *TgbL12m* and *TguL3m* resolved each of them as a part of a macromolecular complex, with a size potentially corresponding to the mitochondrial ribosome (Fig. 2B). This possibility receives further support from the co‐depletion of *Tg*m*S35* upon depletion of *TguS15m* (Fig. 2D), and by the enrichment of mitochondria‐encoded rRNA in the TgmS35 IP fraction (Fig. 2E). These data provide the first direct evidence that, despite the apicomplexan‐fragmented mitochondrial rRNA (Barbrook *et al.*, 2010), the apicomplexan mitochondrial ribosomes are assembled into stable complexes. This is a major step forward in the study of apicomplexan mitochondrial ribosomes which are not well studied and whose composition is not known. While our observation suggests that macro‐molecules containing mitochondrial ribosomal proteins form a stable complex, the question of how they assemble and are held together structurally remains open. A recent study identified novel RNA binding proteins in the apicomplexan mitochondrion and proposed the hypothesis that they may participate in ribosome assembly (Hillebrand *et al.*, 2018). The tagged ribosomes we generated here can be used in the future to try tackling the question of mitochondrial ribosome structure and function in *T. gondii*.

### A new protocol to follow mitochondrial translation in *T. gondii*

The lack of a direct assay to detect mitochondrial translation in Apicomplexa is a major obstacle to study this important pathway. In a recent study, a *P. falciparum* mitochondrial ribosomal protein, the large subunit 13 (which per the new notation (Greber and Ban, 2016) can be named *Pf*uL13m) was characterised (Ke *et al.*, 2018). Like *Tg*mS35, the study found that *Pf*uL13m depletion mutant displayed a growth defect. The authors showed decreased activity of respiratory complex III, whose Cytb subunit is mitochondrially encoded, as a read‐out for translation (Ke *et al.*, 2018). However, the inability to rescue the growth defect using an mETC bypass route available for the *Plasmodium* asexual stages (Ke *et al.*, 2018), complicates the analysis of phenotype specificity. Here, we generated an analytic pipeline that, while still indirect, provides specificity for a translation‐dependent outcome. We show that conditional depletion of *TgmS35* results in disruption of the activity of respiratory complex IV, which contains the mitochondrially encoded subunits (CoxI and CoxIII), at 48 h after gene depletion (Fig. 6B). On the contrary, complex V, for which the subunits are encoded in the nuclear genome, shows no decrease in activity at the 48 h time point (Fig. 6B). Complex V only shows a defect after a long period of *TgmS35* depletion, potentially due to secondary effects (Fig. 6C). We further show that the assembly and activity of the TOM complex is unaffected at the 96 h time point (Fig. 6D and E). This approach of comparing the activities of complexes that do and do‐not depend on mitochondrial translation for their assembly and activity provides a measure of mitochondrial translation in Apicomplexa and can be used in future studies to assess the involvement of other candidates with putative roles in mitochondrial translation, as well as the impact of drugs aimed at mitochondrial translation inhibition. Apicomplexan ribosomal proteins are not well studied. To our knowledge, this is the first functional characterisation of a mitochondrial ribosomal protein in *T. gondii*. Our observations point to mitochondrial translational defect upon *TgmS35* depletion, thus supporting the role of *Tg*mS35 in the translation machinery. Finally, the data provides additional support to an active mitochondrial translation in these parasites.

In model organisms, *in vitro* functional assays performed with isolated mitochondria are extensively used to unravel mitochondrial functions including translation. Previously functional analysis of apicomplexan mitochondria has been hampered by the lack of good quality, large‐scale, organelle enrichment protocols. This led to a reliance on whole cell biochemistry (e.g. Ke *et al.*, 2018; Seidi *et al.*, 2018) and on fluorescence microscopy analysis using fluorescent dyes (e.g. Vercesi *et al.*, 1998; Garbuz and Arrizabalaga, 2017; Ovciarikova *et al.*, 2017) for mitochondrial functional studies. Here, we developed an organelle enrichment protocol which generates high‐quantity fractions enriched with active mitochondria that can be used to investigate the activity of mitochondrial pathways *in vitro*. For example, enriched mitochondria are used for the complex V enzymatic activity performed using native‐PAGE technique (Fig. 6B; Salunke *et al.*, 2018). The protocol we develop here for one of the most tractable apicomplexan parasites can be used to develop additional assays. By this, our work provides an avenue for prioritising mechanistic studies of these key conserved proteins. We found that the use of nitrogen cavitation to lyse *T. gondii* tachyzoites results in optimal lysis (Table S8), while maintaining any of the macromolecular‐complexes examined here intact (TOM, complex IV, complex V and the putative mitochondrial ribosome (Figures 2B, 6B‐D)). Nitrogen cavitation has previously been used for mitochondrial enrichment in *Plasmodium* (Kobayashi *et al.*, 2007; Mather *et al.*, 2010; Hata *et al.*, 2018) and in the unrelated single cell eukaryote Trypanosomes (Schneider *et al.*, 2007). We envisage this protocol will be invaluable for future efforts in the field to study *Toxoplasma* mitochondrial biochemistry, and to characterise other *Toxoplasma* organelles.

## Experimental procedures

### *In silico* search

The mRNA abundance dataset generated by (Behnke *et al.*, 2010) was queried via GeneSpring 11.0. Of the 14 homologs of mitochondrial protein import components (Table S1) 3 do not have cyclical expression pattern (Table S9 – sheet 1). For each of the remaining 11, we searched for other genes with correlating cyclical expression patterns. We ran Euclid and Spearman correlation tests at 0.9 and 0.95 scores and kept lists of correlation that were smaller than 70. This search identified 281 non‐overlapping genes (the original search was performed with release 7.2 of ToxoDB, TGME49 strain; the same group of genes now correspond to 279 predicted genes from this strain) (search steps are shown in Table S9 – sheet 2).

The orthology search leading to the 43 gene list was performed via the ToxoDB link to OrthoMCL. A search strategy queried for *Toxoplasma* genes with orthologs in *Plasmodium* 3D7 and with no orthologs in the three *Cryptosporidium* spp genomes available. The group of TGME49 genes that comply with these criteria was intersected with the 279 group resulting in 43 genes (Table S3 – sheet 2).

Fitness phenotypes scores (Table S6 – columns E‐F) was obtained via the ToxoDB tool. All TGME49 genes were converted to their TGGT1 syntenic orthologs and then sorted according to their fitness scores.

The probability of export to the mitochondria (Table S6 – column B) was assessed using the MitoProt II algorithm (v.1.101) (Claros and Vincens, 1996) (https://ihg.gsf.de/ihg/mitoprot.html).

### Plasmid construct

For ligation‐independent cloning (LIC), between 500bp and 1.5kb homologous region of the 3′ end of each gene of interest (GOI) was amplified by PCR from *T. gondii* RH genomic DNA with LIC sequence flanking these regions (primers are in Table S4). Products were LIC‐cloned into linearised p3HA.LIC.CATΔpac containing a triple HA epitope tag as described previously (Huynh and Carruthers, 2009; Sheiner *et al.*, 2011), or into linearised pTEV.TwinStrep.LIC.CATΔpac or pTEV.TripleFLAG.LIC.CATΔpac, where the triple‐HA was replaced with TEV.TwinStrep or TEV.Triple‐FLAG using In‐Fusion cloning. The vectors were transfected into the TATi∆ku80 line (Sheiner *et al.*, 2011) and selected with chloramphenicol.

For expression of tagged minigenes, the corresponding cDNAs were cloned (primers in Table S4) into the pDT7S4 expression vector that fuses a C‐terminal Myc eptitope tag (van Dooren *et al.*, 2008) via BglII and AvrII restriction sites.

For promoter replacement, the ChopChoP (http://chopchop.cbu.uib.no/) tool was used to identify gRNAs covering the ATG of each GOI. The corresponding gRNAs were cloned into a U6 promoter and CAS9‐GFP expressing vector (Tub‐Cas9‐YFP‐pU6‐ccdB‐tracrRNA) (Curt‐Varesano *et al*., 2016) using the BsaI restriction site, and the final plasmid was purified by DNA midi‐prep (Qiagen) per the manufacturer's protocol. The DHFR selectable cassette and ATc repressible promoter were amplified by PCR from pDT7S4myc (van Dooren *et al.*, 2008; Sheiner *et al.*, 2011). Parasites were transfected with 50 μg of the gRNA/CAS9 vector‐PCR product mixture, and cassette integration was selected with pyrimethamine.

For transient gene disruption, each gene‐specific gRNA (sequences in Table S4) vector, synthesised by GenScript using backbone vector pU6‐SAG1gRNA‐DHFR (Serpeloni *et al.*, 2016), was co‐transfected with the CAS9‐FLAG expressing vector (pU6‐universal) (Sidik *et al.*, 2016).

### Parasites culture

All *T. gondii* lines were cultured on Human Foreskin Fibroblast (HFFs) in DMEM media complemented with 10% FBS, 2% L‐glutamine and 1% Penicillin/Streptomycin antibiotics and grown at 37°C with 5% CO_2_ air atmosphere.

### Stable transfection

Linearised vectors were transfected into TATi∆ku80 (Sheiner *et al.*, 2011) expressing line by electroporation. Parasites were selected by adding the appropriate drug to the culture medium for up to 2 weeks, then cloned on 96 well plates. Positive clones were screened by PCR using the appropriate primers listed in Table S4.

### Immunofluorescence assay and microscopy

Parasites were inoculated onto human foreskin fibroblasts (HFFs) on coverslips for the mentioned time period and fixed with 4% paraformaldehyde (PFA) for 20 min at room temperature (RT). The PFA was washed away thrice with phosphate buffer saline (PBS). Cells were then permeabilised and blocked with blocking buffer (PBS, 0.2% Triton X‐100, 2% Bovine Serum Albumin, BSA) for 20 min at RT. Cells were then labelled with different sets of primary and secondary antibodies: mouse anti‐HA antibody (1:1000, Sigma), rabbit anti‐TgMys (1:1000) (Ovciarikova *et al.*, 2017), mouse anti‐Myc (1:1000, Cell Signalling), rabbit anti‐TgTOM40 (1:2000)(van Dooren *et al.*, 2016); mouse anti‐Strep (1:1000, StrepMAB‐Classic, IBA), mouse anti‐FLAG (1:1000, Monoclonal ANTI‐FLAG® M2, Sigma‐Aldrich) coupled with goat anti‐mouse or anti‐rabbit fluorescent antibody (AlexaFluor 594 or 488 1:1000 (Invitrogen)). Primary antibodies were incubated for 1 h at RT. Coverslips were then washed thrice for 5 min with PBS‐0.2% Triton X‐100 and incubated with secondary antibodies for 45 min at RT in the dark. Coverslips were washed as above and mounted on microscope slides using DAPI‐fluoromountG (Southern Biotech). Images were taken using a Delta Vision microscope as described (Ovciarikova *et al.*, 2017).

### Mitochondrial morphology scoring

*CRISPR/Cas9 transient expression* – Parasites transiently co‐expressing CAS9‐FLAG expressing vector (pU6‐universal) (Sidik *et al.*, 2016) and gene‐specific sgRNA containing vector (Serpeloni *et al.*, 2016), for genes *TGME49_214790*/ *263680* and *226280,* were fixed 48 h post‐transfection. The CAS9‐FLAG and mitochondria were visualised by immunofluorescence as described above. 50 vacuoles of CAS9‐FLAG expressing parasites for each gene were then counted and mitochondrial morphology was assessed as followed: mitochondrial morphologies were sorted into two categories: (a) ‘normal’ mitochondrial morphology (open, sperm‐like morphologies (Ovciarikova *et al.*, 2017)), (b) abnormal morphology (anything other than open and sperm‐like morphologies), then scored. Dead parasites (displaying one or two balled mitochondria (Ovciarikova *et al.*, 2017)) were not considered. Experiments were performed in triplicate.

*Conditional knockdown* – *rTgmS35*‐3xHA mutant line was grown in the presence or absence of ATc 0.5 µM for 48 h, fixed and mitochondria were visualised by immunofluorescence using anti‐TgMys, as described above. Mitochondrial morphology was scored as ‘normal’ (vacuoles that have majority full lasso) or ‘abnormal’ (vacuoles with majority sperm shape of otherwise abnormal). Replicating parasites were excluded. Experiments were performed in triplicate, and a Chi‐squared statistical test was performed.

### Plaque assay

About 1000, 300 and 100 parasites were inoculated in 6‐well plates in duplicates, in the presence or absence of ATc 0.5 µM for 9 days. Cells were then fixed with 100% methanol for 30 min at RT, washed with PBS and crystal violet dye was added for 2 h at RT to stain host cells, and then washed with PBS.

### Western blot

Samples were resuspended in 1X NuPAGE LDS loading dye (Invitrogen) with 5% v/v beta‐mercaptoethanol, then boiled at 95°C for 5 min and separated by SDS‐PAGE. Proteins were transferred under wet conditions (in Towbin buffer (0.025 M TRIS 0.192 M Glycine 10 % Methanol) for 60 min at 100 V) to nitrocellulose membrane (0.45 μm Protran™). Blots were labelled with the appropriate set of antibodies: primary rat anti‐HA (1:500, Sigma), mouse anti‐Myc (1:1000, Cell signalling), mouse anti‐Strep (1:4000, StrepMAB‐Classic HRP conjugate, IBA), mouse anti‐FLAG (1:1000, Monoclonal ANTI‐FLAG® M2, Sigma‐Aldrich) or rabbit anti‐TgTOM40 (1:2000; van Dooren *et al.*, 2016) antibodies coupled to secondary horseradish peroxidase (HRP) (Promega for mouse and rabbit, Abcam for rat) conjugated antibodies (1:10,000) or secondary fluorescent antibodies IRDye® 800CW (1:10000, LIC‐COR), and visualised using the Pierce ECL Western Blotting Substrate (Thermo Scientific) or the Odyssey LCX respectively.

For the blue native‐PAGE, proteins were transferred onto a PVDF membrane (0.45 μm, Hybond™) using wet transfer in Towbin buffer (0.025 M TRIS 0.192 M Glycine 10 % Methanol) for 60 min at 100 V. After transfer and immunolabelling, visualisation was carried out as described above with Pierce ECL Western Blotting Substrate.

### Protein import assay

Parasites were transfected with the Hsp60_L_‐mDHFR‐cMyc vector (van Dooren *et al.*, 2016) (kind gift from Giel van Dooren), after growth in the presence or absence of 0.5 µM ATc for 24 and 72 h. Transfected parasites were collected after an additional 24 h of growth in their respective treatment and western blot was performed using the respective parasite pellets as described above. Band intensity was measured using the ImageJ software and the ratio between the mature and pre‐processed band intensity was calculated. A one‐way ANOVA statistical test was performed.

### Preparation of mitochondria‐enriched fraction

Parasites were cultured on HFFs in T150 flasks in the presence or absence of 0.5 µM ATc. Egressed parasites were collected (or, where necessary, parasites were scraped and syringed, e.g. at 96 h) and filtered through a 3 µm pore size membrane. From this point on, all steps are carried out at 4°C. Parasites were centrifuged at 1500 × g for 15 min. Media was discarded and pellets were resuspended in PBS. Parasites in PBS were counted on a Neubauer chamber and centrifuged at 1500 × g for 15 min. The supernatant was discarded and the pellet resuspended in lysis buffer (50 mM HEPES‐KOH pH 7.4, 210 mM mannitol, 70 mM sucrose, 1 mM EGTA, 5 mM EDTA, 10 mM KCl, 1 mM DTT, 1 protease inhibitor cocktail tablet (Complete Mini, EDTA‐free; Roche) per 50 ml) to a concentration of 5 × 10^8^ parasites ml^−1^. Parasites were transferred into a precooled nitrogen cavitation chamber and incubated at a pressure of 2750psi for 15 min on ice. After pressure release and centrifugation at 1500 × g for 15 min, the supernatant (lysate) was kept, and the pellet, containing unbroken parasites, was resuspended again at 5 × 10^8^ parasites ml^−1^ in lysis buffer. Repeating rounds of nitrogen cavitation were performed until >95% of parasite lysis was achieved (evaluated via counting using Neubauer chamber). Pooled lysates were spun down at 1500 × g to remove any remaining unbroken parasites. The lysate was then aliquoted to the desired amount in microcentrifuge tubes and span down at 16,000 × g for 25 min. Supernatant discarded and pellet used immediately or stored at −80°C until use.

### Blue‐native polyacrylamide gel electrophoresis

Whole parasite or previously aliquoted enriched mitochondria fraction were mixed with solubilisation buffer (750 mM aminocaproic acid, 50 mM Bis‐Tris–HCl pH 7.0, 0.5 mM EDTA, 1 % (w/v) dodecyl maltoside) and incubated on ice for 5 min. The mixture was centrifuged at 16,000 × g at 4°C for 10 min. The supernatant containing solubilised membrane proteins was transferred into a new microcentrifuge tube and 0.25% (w/v) Coomassie G250 (final concentration) was added. The anode buffer (50 mM Bis‐Tris–HCl pH 7.0) and cathode buffer (50 mM Tricine, 15 mM Bis‐Tris–HCl pH 7.0, 0.02% Coomassie G250 (Serva)) were poured into their respective tank compartment and the appropriate amount of protein (55 μg or 5x10^5^ parasites) was loaded per lane on a NativePAGE™ 4–16% or 3–12% Bis‐Tris Gel (Novex‐ Life technologies). 5 μl NativeMark™ (Invitrogen) was used as a molecular weight marker. Gels were run for ~45 min at 100 V, 4–10 mA at 4°C with cathode buffer containing 0.02% (w/v) Coomassie G250, then for ~2.5 h at 250 V, 15 mA with cathode buffer containing 0.002% (w/v) Coomassie G250, until the dye front reached the bottom of the gel.

### High‐resolution clear‐native polyacrylamide gel electrophoresis

Carried out as described in (Wittig *et al.*, 2007) with minor modifications. Briefly, whole parasite (3 × 10^7^) or previously aliquoted mitochondria enriched fraction (75 µg) were mixed with solubilisation buffer (50 mM NaCl, 50 mM Imidazole, 2 mM 6‐aminohexanoic acid, 1 mM EDTA – HCl pH 7.0, 2% (w/v) n‐dodecylmaltoside) and incubated on ice for 10 min. The mixture was centrifuged at 16,000 × g at 4°C for 15 min. A final concentration of 6.25% glycerol and 0.125% Ponceau S was added to the solubilised membrane proteins. The anode buffer is composed of 25 mM Imidazole – HCl pH to 7.0 and the cathode buffer of 50 mM Tricine, 7.5 mM Imidazole, 0.02% w/v n‐dodecylmaltoside, 0.05% sodium deoxycholate. Gels were run for 30 min at 100 V, 10 mA, then 250–300 V, 15 mA at 4°C until it the dye front reached the bottom.

### In‐gel activity staining

Activity stains were carried out as described previously (Sabar *et al.*, 2005). Briefly, gels were equilibrated in buffer without staining reagents for 10 min. For complex IV, oxidation activity was shown using 50 mM KH_2_PO_4_, pH 7.2, 1 mg ml^−1^ cytochrome *c*, 0.1% (w/v) 3,3′‐diaminobenzidine tetrahydrochloride. Stains were visible after 30 min. Staining was continued with pictures taken of the stained gels at regular intervals. For complex V, ATP hydrolysis activity was visualised using 35 mM Tris, 270 mM glycine, pH 8.4, 14 mM MgCl_2_, 11 mM ATP, 0.3% (w/v) Pb(NO_3_)_2_. Stains were visible after 1 h. Pictures were taken against a black background for optimal visualisation of white lead precipitates.

### Mass spectrometry

Proteins were identified using nanoflow HPLC electrospray tandem mass spectrometry (nLC‐ESI.MS/MS) at Glasgow Polyomics. Tryptic peptides, generated using in‐gel digest procedure (Williams *et al.*, 2018), were analysed as previously described (Akpunarlieva *et al.*, 2017). Protein identities were assigned using the Mascot search engine (v2.6.2, Matrix Science) to interrogate protein sequences in the *T. gondii* genome sequence dataset, ToxoDB 35 release. During result analysis, only peptides with Mascot score of 20 and above (namely the probability that this match might be a random event is 10^−2^ or lower) were included in the analysis.

### Immunoprecipitation, RNA extraction and RT‐PCRs

Parasites were gown in HFF in T175 flasks until lysed (2–3 × 10^8^ cells), then collected. Dry pellets were stored at −80°C until day of the experiment. Pellets were lysed on ice for 15 min in TBS containing 1% DDM (Thermso Fisher), 0.4 U/μl of RNaseOUT™ Recombinant Ribonuclease Inhibitor (Thermo Fisher) and 1 mM DTT (Sigma). Lysates were spun at 16000 × g 10 min at 4°C. Supernatants were transferred to microcentrifuge tubes containing 50 μl of washed Pierce® Anti‐HA Agarose beads (Thermo scientific). The immunoprecipitation was performed according to the manufacturer's instruction. Elution was performed using 0.1 M glycine, pH 2.6. RNA extraction was performed using the RNeasy Mini kit (Qiagen) with DNase I treatment (Thermo Fisher). Reverse transcription reaction used the High Capacity RNA‐to‐cDNA Kit (Thermo Fisher). PCR used RT‐outcome as template, and primers for mito‐RNA, api‐RNA and actin, and was performed with annealing temperature of 51°C and elongation time of 25 s for 30 cycles.

## Author contributions

L.S. conceived the research; L.S. A.L. and A.E.M developed the methods; A.L. A.E.M. J.O. J.T. A.M P.F and L.S. designed and performed the experiments; L.S. A.L. and A.E.M. discussed the data and wrote the paper.
