# Supplementary material for: Identification of the Toxoplasma gondii mitochondrial ribosome, and characterisation of a protein essential for mitochondrial translation
Source: Mol Microbiol. 2019 Jul 24;112(4):1235–52. doi: 10.1111/mmi.14357 (PMC6851545; doi:10.1111/mmi.14357)
Supplement: Supplementary file 1 [file MMI-112-1235-s001.pdf]

Figure S1

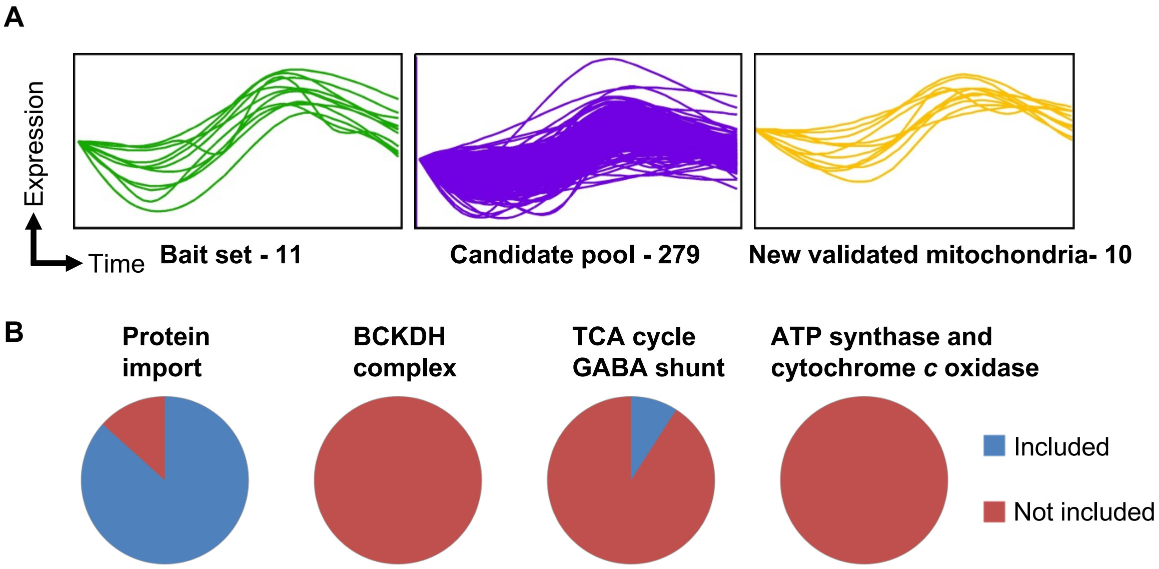

A
Expression
Time
Bait set - 11
Candidate pool - 279
New validated mitochondria- 10
B
Protein import
BCKDH complex
TCA cycle GABA shunt
ATP synthase and cytochrome *c* oxidase
Included
Not included

Figure S2

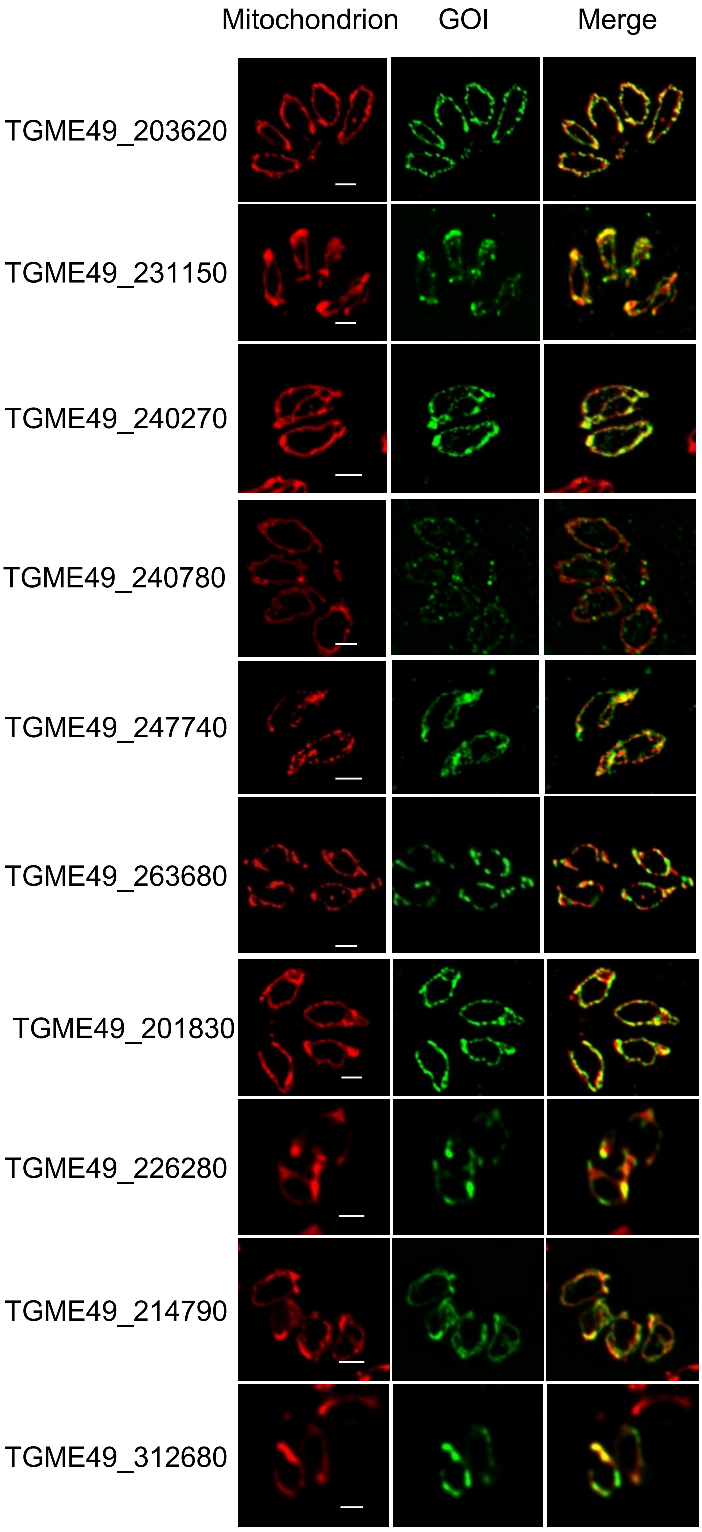

Mitochondrion
GOI
Merge
TGME49_203620
TGME49_231150
TGME49_240270
TGME49_240780
TGME49_247740
TGME49_263680
TGME49_201830
TGME49_226280
TGME49_214790
TGME49_312680

Figure S3

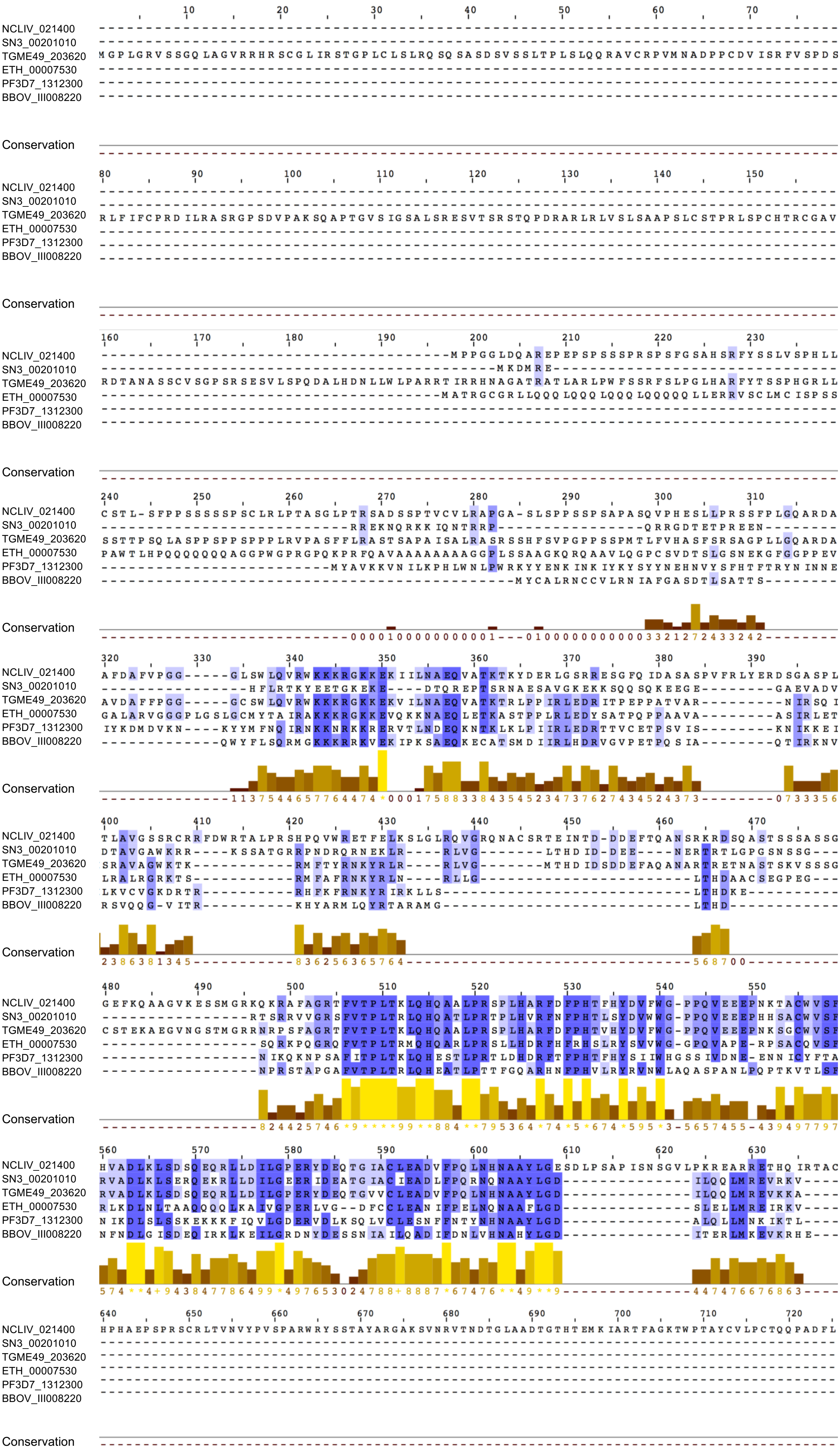
NCLIV_021400
SN3_00201010
TGME49_203620
ETH_00007530
PF3D7_1312300
BBOV_III008220
Conservation

Fig. S4

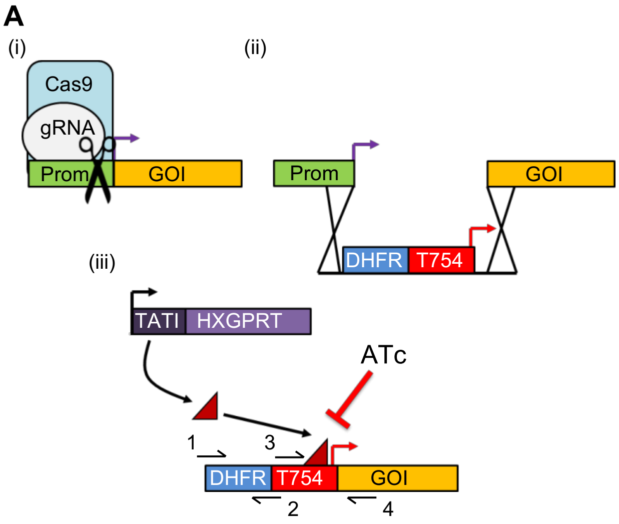
A
(i)
Cas9
gRNA
Prom
GOI
(ii)
Prom
GOI
DHFR
T754
(iii)
TATI
HXGPRT
ATc
1
3
DHFR
T754
GOI
2
4

Figure S5

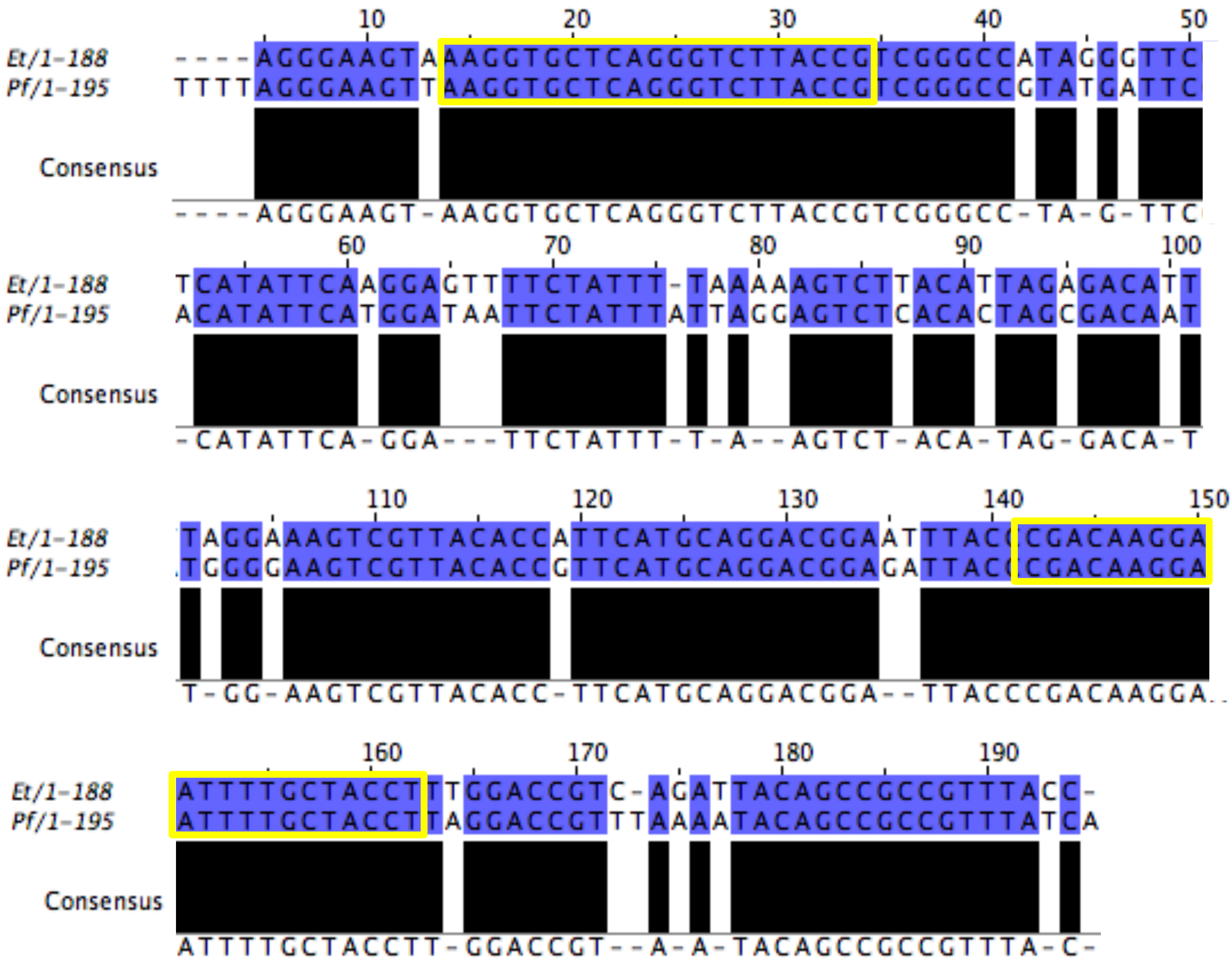

Figure S6

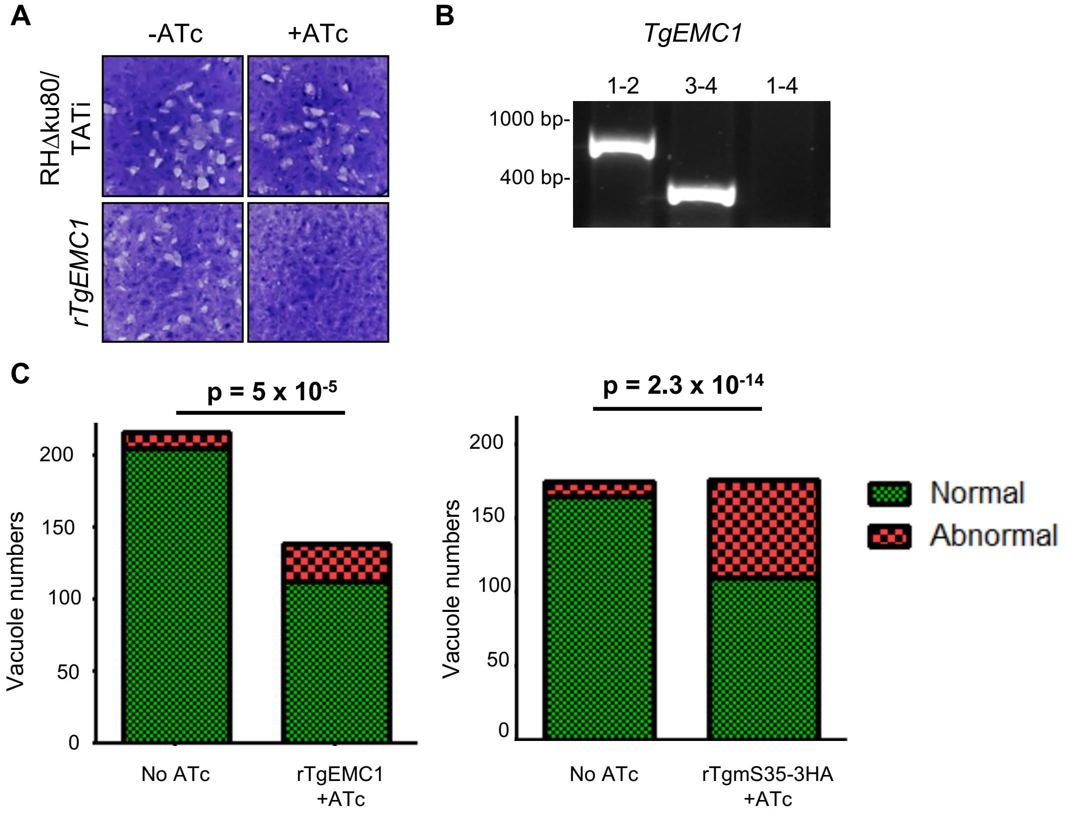
A
-ATc
+ATc
RHΔku80/ TATi
rTgEMC1
B
TgEMC1
1-2
3-4
1-4
1000 bp-
400 bp-
C
p = 5 x 10-5
p = 2.3 x 10-14
Vacuole numbers
200
150
100
50
0
No ATc
rTgEMC1 +ATc
rTgmS35-3HA +ATc
Normal
Abnormal

Fig. S7

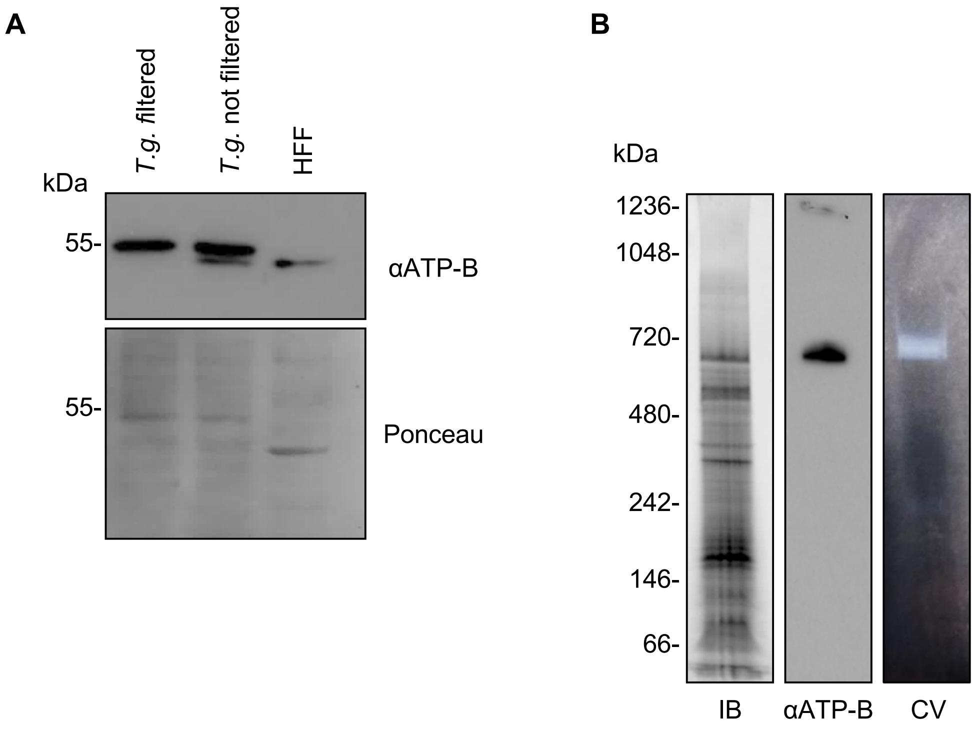
A
T.g. filtered
T.g. not filtered
HFF
kDa
55-
αATP-B
55-
Ponceau
B
kDa
1236-
1048-
720-
480-
242-
146-
66-
IB
αATP-B
CV
