# Supplementary material for: Identification of the Toxoplasma gondii mitochondrial ribosome, and characterisation of a protein essential for mitochondrial translation
Source: Mol Microbiol. 2019 Jul 24;112(4):1235–52. doi: 10.1111/mmi.14357 (PMC6851545; doi:10.1111/mmi.14357)
Supplement: Supplementary file 2 [file MMI-112-1235-s002.docx]

Supplementary tables

**Table S1 – *Toxoplasma* genes encoding components of the mitochondrial protein import pathway**

| 14 genes used for mining of the cyclical mRNA expression data^±^ | | | | |
| --- | --- | --- | --- | --- |
| Protein name | Gene ID of *Plasmodium** | GeneID at time of search (2012) | Current *T. gondii* homolog ID | Recovered in search? |
| Tim23 | PF13_0300 | TGME49_014150 | TGME49_214150 | Y |
| Tim17 | PF14_0328 | TGME49_112220 | TGME49_312220 | Y |
| Tim50 | PF07_0110 | TGME49_083590 | TGME49_283590 | Y |
| Tim44 | PF11_0265 | TGME49_027830 | TGME49_227830 | Y |
| GrpE | PF11_0258 | TGME49_065220 | TGME49_265220 | Y |
| Pam18 | PF07_0103 | TGME49_002810 | TGME49_202810 | N |
| Tom40 | PFF0825c | TGME49_018280 | TGME49_218280 | Y |
| Tim 8 | PFL2065c / PF14_0208 | TGME49_054610 | TGME49_254610 | Y |
|  |  | TGME49_074090 | TGME49_274090 | Not used^ |
| Tim9 | PF13_0358 | TGME49_015390 | TGME49_215390 | Y |
|  |  | TGME49_060850 | TGME49_260850 | Not used^ |
| Tim 22 | PFF1330c | TGME49_025710 | TGME49_225710 | Y |
| HSP70 | PF11_0351 | TGME49_051780 | TGME49_251780 | Not used^ |
| Erv1 | PF10_0029 | TGME49_032820 | TGME49_232815 | Y |
|  |  |  |  |  |
| Additional homologs of mitochondrial import proteins not used as bait | | | | |
| Cpn10 |  |  | TGME49_263530** | Y |
| GroS |  |  | TGME49_273960** | N |
| Sam50 |  |  | TGME49_205570** | N |
| Pam16 |  |  | TGME49_249910** | V |
| Tim10 | PFL0430w |  | TGME49_215390** | Y |
| Tom22 | PFE1230c |  | Null | N/A |

± From: Coordinated progression through two subtranscriptomes underlies the tachyzoite cycle of *Toxoplasma gondii*. Behnke MS, Wootton JC, Lehmann MM, Radke JB, Lucas O, Nawas J, Sibley LD, White MW. PLoS One. 2010 Aug 26;5(8):e12354.

* From: Metabolic maps and functions of the *Plasmodium* mitochondrion. van Dooren GG, Stimmler LM, and McFadden GI

^mRNAs of these genes did not show cyclical expression thus they were no used as baits.

** From: The Import of Proteins into the Mitochondrion of *Toxoplasma* *gondii*. van Dooren GG, Yeoh LM, Striepen B, McFadden GI

**Table S2 – Lists of genes resulting from searches based on mRNA expression patterns and on phylogenetic distribution**

Separate Excel file

**Table S3 – Genes from the *in silico* search whose localization was experimentally addressed in this work**

| Gene ID | Predicted product (TGME49) | Localisation  Form of tagging | MitoProt | Predotar | Proximity tagging proteome | Phenotype score |
| --- | --- | --- | --- | --- | --- | --- |
| TGME49_203620^§^ | hypothetical protein | Mitochondrial  cDNA | 0.9734 | 0.53 | no* | -4.66 |
| TGME49_226270 | hypothetical protein | Apicoplast  endogenous | 0.0402 | 0.01 | no | 0.23 |
| TGME49_231150 | hypothetical protein | Mitochondrial  endogenous | 0.9986 | 0.33 | yes | -5.21 |
| TGME49_240270^§^ | hypothetical protein | Mitochondrial  cDNA | 0.2831 | 0.04 | no* | -4.65 |
| TGME49_240780 | hypothetical protein | Mitochondrial  endogenous | 0.3871 | 0.76 | no | -3.95 |
| TGME49_247740 | hypothetical protein | Mitochondrial  endogenous | 0.0647 | 0.03 | no* | 0.31 |
| TGME49_257110 | hypothetical protein | no signal  cDNA | 0.6373 | 0.01 | no | -3.21 |
| TGME49_263680 | hypothetical protein | Mitochondrial  endogenous | 0.9490 | 0.52 | no* | -4.30 |
| TGME49_310500 | hypothetical protein | no signal  cDNA | 0.9211 | 0.11 | no* | -4.10 |
| TGME49_201830 | ribosomal protein L37 | Mitochondrial  endogenous | 0.9991 | 0.43 | no | -4.94 |
| TGME49_226280 | ribosomal protein L28, putative | Mitochondrial  endogenous | 0.9971 | 0.65 | yes | -4.91 |
| TGME49_214790^§^ | glycoprotein | Mitochondrial  endogenous | 0.8383 | 0.47 | yes | -3.48 |
| TGME49_311750 | mago binding protein | Cytosol  cDNA | 0.1980 | 0.01 | no | -0.99 |
| TGME49_312680 | 60S ribosomal protein L27, putative | Mitochondrial  cDNA | 0.9388 | 0.14 | no | -1.34 |

Mitochondrial targeting predictions are done using MitoProt II (<https://ihg.gsf.de/ihg/mitoprot.html>) and Predotar (<https://urgi.versailles.inra.fr/predotar/)>. The presence (yes) or absence (no) of the gene product in the proximity tagging based mitochondrial proteome is shown (Seidi et al., 2018) (no - no mass spectrometry data; no* - mass spectrometry data found but the gene is not included in the final list of 421 genes). The fitness score is based on the whole genome CRISPR/CAS9 screen (Sidik et al., 2016) (geneIDs in bold and with ^§^ - essentiality/importance for fitness confirmed here).

**Table S4 – Primers and gRNA sequences used in this study**

| Experiment | Gene ID | Details | Sequence |
| --- | --- | --- | --- |
| Localisation | TGME49_203620 | Endogenous HA tag_Forward | TACTTCCAATCCAATTTAATGCCCAACAAGAGTGGCTGT |
|  | TGME49_203620 | Endogenous HA tag_reverse | TCCTCCACTTCCAATTTTAGCCGCCTTCTTCACCTCTCGC |
|  | TGME49_312680 | cDNA-Myc_Forward | ctatAGATCTATGTCTCTGCTCTCTCTCTCGGTGCCG |
|  | TGME49_312680 | cDNA-Myc_reverse | gtatCCTAGGGAAGTCTTCTCCATCATCCAACTCCCG |
|  | TGME49_226280 | cDNA-Myc_Forward | gggcAGATCTATGCCGAAGAATCTTAACTTGTGG |
|  | TGME49_226280 | cDNA-Myc_reverse | gtatCCTAGGAAAGGCACGGTTTTCCCGGTACTTGTG |
|  | TGME49_231150 | Endogenous HA tag_Forward | TACTTCCAATCCAATTTAATGCCGCCGATCGACGTCGTTCTGC |
|  | TGME49_231150 | Endogenous HA tag_reverse | TCCTCCACTTCCAATTTTAGCCAGAACCCAGAAGTGTGCGCC |
|  | TGME49_240780 | Endogenous HA tag_Forward | TACTTCCAATCCAATTTAATGCATCATCACGGTTGGTAAGG |
|  | TGME49_240780 | Endogenous HA tag_reverse | TCCTCCACTTCCAATTTTAGCGATCTGAGAGTCTCTGGGGAC |
|  | TGME49_247740 | Endogenous HA tag_Forward | TACTTCCAATCCAATTTAATGCGGAGGGTGTAGGGTTCATGCCC |
|  | TGME49_247740 | Endogenous HA tag_reverse | TCCTCCACTTCCAATTTTAGCGGAGTCTCCGAGTTCTGGAAGC |
|  | TGME49_263680 | Endogenous HA tag_Forward | TACTTCCAATCCAATTTAATGCGTTCAGTCTCCTGGAGGCCTTTTCG |
|  | TGME49_263680 | Endogenous HA tag_reverse | TCCTCCACTTCCAATTTTAGCGCGCTGTTTCAAGGTGAAGGGG |
|  | TGME49_270150 | Endogenous HA tag_Forward | tacttccaatccaatttaatGCGCCTCCCAAACGGGACTCC |
|  | TGME49_270150 | Endogenous HA tag_reverse | tcctccacttccaattttagcCGAGAGATTTGTGAGACTTCG |
|  | TGME49_201830 | Endogenous HA tag_Forward | tacttccaatccaatttaatGCGGTCTCCCAAGACACTCCG |
|  | TGME49_201830 | Endogenous HA tag_reverse | tcctccacttccaattttagcGGCATCCCAGAAAAGCGGCTTCAC |
|  | TGME49_214790 | Endogenous HA tag_Forward | TACTTCCAATCCAATTTAATGCATGCGGGGATTCCAAGAAACTCGG |
|  | TGME49_214790 | Endogenous HA tag_reverse | TCCTCCACTTCCAATTTTAGCGTGTTCGACGAACTGAGCGAG |
|  | TGME49_ 051950 | Endogenous TwinStrep_Forward | tacttccaatccaatttaat |
|  | TGME49_ 051950 | Endogenous TwinStrep_reverse | gaattcccgtcctccacttccaattttaGC |
|  | TGME49_230050 | Endogenous TripleFLAG_Forward | tacttccaatccaatttaat |
|  | TGME49_230050 | Endogenous TripleFLAG _reverse | gaattcccgtcctccacttccaatttta |
| Transient disruption via CRISPR/ Cas9 | TGME49_214790 | gRNA forward primer | GCTCTGCGGTGCTGCGCGAG |
|  | TGME49_226280 | gRNA forward primer | GGGGAAGTACCTGCGGCTGG |
|  | TGME49_263680 | gRNA forward primer | GTCCTCCACCCCAAGACGGT |
| Promoter replacement mediated via CRISPR/ Cas9 | TGME49_203620 | gRNA sequence | GAAGACAGCATGGGGCCACTTGG |
|  | TGME49_203620 | gRNA forward primer | aagttGAAGACAGCATGGGGCCACTg |
|  | TGME49_203620 | gRNA reverse primer | aaaacAGTGGCCCCATGCTGTCTTCa |
|  | TGME49_203620 | Homology region_forwrad | GACATTAAAGGCAACCACAAGAACAGAGAACATCACAGTGAGAAGACAGCaagcttcgccaggctgtaaatcc |
|  | TGME49_203620 | Homology region_reverse | CGACGGACCCCAGCAAGCTGGCCCGAGGACACCCGGCCAAGTGGCCCCATtggttgaagacagacgaaagcagttg |
|  | TGME49_203620 | Screening test_forward | CTTTTCTGGCCTCTGCCTCAGC |
|  | TGME49_203620 | Screening test_reverse | CCGAGAAAGCGCGGAGCCAATA |
|  | TGME49_016040 | gRNA sequence | GAATGGCTTCTCTGGCGCGTCGG |
|  | TGME49_016040 | gRNA forward primer | aagttGAATGGCTTCTCTGGCGCGTg |
|  | TGME49_016040 | gRNA reverse primer | aaaacACGCGCCAGAGAAGCCATTCa |
|  | TGME49_016040 | Homology region_forwrad | gtcgtctcctccttcgtcttcttctgcttcttcttctctcttcaagtggaaagcttcgccaggctgtaaatcc |
|  | TGME49_016040 | Homology region_reverse | GCCCCCAACGCCTTGGTGGGCGAGAAAATCCGACGCGCCAGAGAAGCCATtggttgaagacagacgaaagcagttg |
|  | TGME49_016040 | Screening test_forward | CAGCTTCAAGTCAACTTGCC |
|  | TGME49_016040 | Screening test_reverse | GTCTAAACACCATGCAGCTG |
|  | TGME49_205740 | gRNA forward primer | aagttGTCTGTTCCACAAGGTAGTAg |
|  | TGME49_205740 | gRNA reverse primer | aaaacTACTACCTTGTGGAACAGACa |
|  | TGME49_205740 | Homology region_forwrad | GGAAAGGCTGCCGCTTGTCGCTGTCGCTTGAGTCTGTTCCACAAGGTAGTaagcttcgccaggctgtaaatcc |
|  | TGME49_205740 | Homology region_reverse | CAGACCTTTCGGAGGGGAACAGGTTCATCCACTAGGTAAGAAGGCGCCATtggttgaagacagacgaaagcagttg |
|  | TGME49_205740 | Screening test_forward | CCACTTCCACCACTCATCCTTA |
|  | TGME49_205740 | Screening test_reverse | CGTGACGTCGGCCACCTGT |
|  |  | DHFR cassette test_reverse | CACGGTTATCAAACCCGAG |
|  |  | DHFR cassette test_forward | CGGTTCGCTTGAAGAAGG |
|  | TGME49_240270 | gRNA sequence | GTCTCTTCTGCTTTTCGCCATGG |
|  | TGME49_240270 | gRNA forward primer | aagttGTCTCTTCTGCTTTTCGCCAg |
|  | TGME49_240270 | gRNA reverse primer | aaaacTGGCGAAAAGCAGAAGAGACa |
|  | TGME49_240270 | Homology region_forwrad | TTTCTCCCCGGCTTTCGCGCTTTTCGCTTCTGTCTCTTCTGCTTTTCGCCaagcttcgccaggctgtaaatcc |
|  | TGME49_240270 | Homology region_reverse | TGGCGCTGCTTGCACTTGTGCTTGCTGCGCTTCCCCTTCGGCACCGCCATtggttgaagacagacgaaagcagttg |
|  | TGME49_240270 | Screening test_forward | ATCATTTCTCTCGTTCTCTCACC |
|  | TGME49_240270 | Screening test_reverse | gctgtccttcctcttcttcaga |
| qRT-PCR | TGME49_203620 | Examine expression | AACCAAGCGCATGTTTACGT |
|  | TGME49_203620 | Examine expression | AGTCGAACCGTTCACTCCTT |
| Mitochondria encoded rRNA | Mitochondrial rRNA fragment | Examine presence in IP fraction | aaggtgctcagggtcttaccg |
|  | Mitochondrial rRNA fragment | Examine presence in IP fraction | aggtagcaaaattccttgtcg |

**Table S5 – Complex IV components identification from band in complex IV enzymatic assay, by mass spectrometry**

| Accession | Name* | Experiment 1 | | | Experiment 2 | | |
| --- | --- | --- | --- | --- | --- | --- | --- |
|  |  | **Score** | **No. of significant matches** | **No. of significant sequences** | **Score** | **No. of significant matches** | **No. of significant sequences** |
| TGGT1_226590 | *Tg*Cox2a | 222 | 9 | 3 | 152 | 5 | 2 |
| TGGT1_310470 | *Tg*Cox2b | 257 | 7 | 3 | 155 | 3 | 2 |
| TGGT1_209260 | *Tg*Cox5b | 593 | 20 | 10 | 289 | 11 | 7 |
| TGGT1_254030 | *Tg*ApiCox13 | 36 | 1 | 1 | N/A | N/A | N/A |
| TGGT1_242840 | *Tg*ApiCox14 | 155 | 5 | 3 | 36 | 1 | 1 |
| TGGT1_265370 | *Tg*ApiCox16 | 185 | 6 | 4 | 97 | 2 | 2 |
| TGGT1_221510 | *Tg*ApiCox18 | 151 | 6 | 3 | 143 | 4 | 3 |
| TGGT1_247770 | *Tg*ApiCox19 | 281 | 16 | 4 | 162 | 8 | 5 |
| TGGT1_262640 | *Tg*ApiCox23 | 148 | 6 | 4 | 40 | 1 | 1 |
| TGGT1_286530 | *Tg*ApiCox24 | 151 | 4 | 3 | 80 | 2 | 2 |
| TGGT1_264040 | *Tg*ApiCox25 | 545 | 15 | 8 | 546 | 11 | 7 |
| TGGT1_306670 | *Tg*ApiCox26 | 388 | 14 | 5 | 130 | 7 | 5 |
| TGGT1_297810 | *Tg*ApiCox30 | 526 | 15 | 10 | 432 | 14 | 9 |
| TGGT1_229920 | *Tg*ApiCox35 | 564 | 21 | 10 | 265 | 9 | 7 |
| TGME49_237120 | *Tg*CoxIII | 67 | 1 | 1 | 57 | 1 | 1 |

*Name according to (Seidi *et al*., 2018).

**Table S6 – *In silico* analysis of features of the genes in the 279 dataset: phylogenetic distribution, essentiality scores, number of TMDs and predicted localization**

Separate Excel file

**Table S7 – All putative *Toxoplasma* mitoribosomal proteins with their new notation and with experimental validation of mitochondrial localisation where available**

| Old Name | New Name | Gene ID | SSU/LSU | This study? | Mitoprot | CRISPR score | Proximity tagging proteome |
| --- | --- | --- | --- | --- | --- | --- | --- |
| RPS5 | uS5m | TGME49_285970 | SSU | No | 0.9901 | -4.01 | Yes |
| RPS6 | bS6m | TGME49_284660 | SSU | No | 0.9850 | -2.47 | No |
| RPS8 | uS8m | TGME49_261380 | SSU | No | 0.5097 | -3.22 | No* |
| RPS9 | uS9m | TGME49_207940 | SSU | No | 0.2581 | -5.14 | No* |
| RPS11 | uS11m | TGME49_231120 | SSU | No | 0.8953 | -3.39 | No* |
| RPS12 | uS12m | TGME49_219770 | SSU | No | 0.9978 | -4.28 | No* |
| RPS14 | uS14m | TGME49_277700 | SSU | No | 0.3127 | -4.37 | Yes |
| RPS15 | uS15m | TGME49_216040 | SSU | Yes | 0.9885 | -4.51 | Yes |
| RPS16 | bS16m | TGME49_318420 | SSU | No | 0.9771 | -3.98 | No |
| RPS17 | uS17m | TGME49_273060 | SSU | No | 0.9506 | -4.29 | No |
| RPS18 | bS18m | TGME49_222990 | SSU | No | 0.9725 | -3.84 | Yes |
| RPS22 | mS22 | TGME49_310710 | SSU | No | 0.9594 | -4.01 | No* |
| RPS29 | mS29 | TGME49_310118* | SSU | No | 0.0510 | -5.58 | Yes |
| RPS35 | mS35 | TGME49_203620 | SSU | Yes | 0.9734 | -4.66 | No* |
| RPL1 | uL1m | TGME49_244580 | LSU | No | 0.0032 | -3.21 | No |
| RPL3 | uL3m | TGME49_230050 | LSU | Yes | 0.8966 | -4.50 | Yes |
| RPL4 | uL4m | TGME49_223660 | LSU | No | 0.9945 | -3.06 | Yes |
| RPL7/L12 | bL12m | TGME49_251950 | LSU | Yes | 0.9672 | -3.60 | Yes |
| RPL11 | uL11m | TGME49_254380 | LSU | No | 0.4201 | -3.52 | No* |
| RPL13 | uL13m | TGME49_225240 | LSU | No | 0.1743 | -3.34 | Yes |
| RPL14 | uL14m | TGME49_225250 | LSU | No | 0.9664 | -3.94 | No |
| RPL15 | uL15m | TGME49_253800 | LSU | No | 0.9980 | -1.91 | Yes |
| RPL16 | uL16m | TGME49_220150 | LSU | No | 0.2185 | -2.48 | No* |
| RPL17 | bL17m | TGME49_297170 | LSU | No | 0.9926 | -4.67 | No* |
| RPL19 | bL19m | TGME49_313960 | LSU | No | 0.0246 | -4.44 | Yes |
| RPL20 | bL20m | TGME49_293310 | LSU | No | 0.4490 | -4.56 | No |
| RPL21 | bL21m | TGME49_202350 | LSU | No | 0.8661 | -5.07 | Yes |
| RPL22 | uL22m | TGME49_310490 | LSU | No | 0.8839 | -5.95 | No* |
| RPL23 | uL23m | TGME49_260660 | LSU | No | 0.8839 | -2.83 | Yes |
| RPL24 | uL24m | TGME49_216010 | LSU | No | 0.4920 | -3.70 | Yes |
| RPL27 | bL27m | TGME49_263110 | LSU | No | 0.8760 | -4.63 | No* |
| RPL28 | bL28m | TGME49_226280 | LSU | Yes | 0.9971 | -4.91 | Yes |
| RPL29 | uL29m | TGME49_263550 | LSU | No | 0.9585 | -3.59 | No* |
| RPL33 | bL33m | TGME49_308930 | LSU | No | 0.9481 | -1.69 | No* |
| L2 | uL2m | TGME49_254400 | LSU | No | 0.9240 | -5.44 | Yes |
| L9 | bL9m | TGME49_214870 | LSU | No | 0.9776 | -2.04 | Yes |
| L25 | bL25m | TGME49_314295 | LSU | No | 0.9999 | -4.48 | Yes |
| L51 | mL43 | TGME49_289140 | LSU | No | 0.8901 | -1.90 | Yes |
| - | mL41 | TGME49_312680 | LSU | Yes | 0.9388 | -1.34 | No |
| - | mL54 | TGME49_201830 | LSU | Yes | 0.9991 | -4.94 | No |

**Table S7 – Putative mitoribosomal proteins in *Toxoplasma gondii*.** Putative mitoribosomal proteins identified from Gupta *et al*., 2014; Seidi *et al*., 2018 and this study. New names were tentatively assigned based to the new nomenclature for ribosomal proteins (Ban *et al*., 2014; Greber and Ban, 2015). New mitochondrial targeting predictions are done using MitoProt II (<https://ihg.gsf.de/ihg/mitoprot.html>). The presence (yes) or absence (no) of the gene product in the proximity tagging based mitochondrial proteome is shown (Seidi *et al*., 2018) (no - no mass spectrometry data; no* - mass spectrometry data found but the gene is not included in the final list of 421 genes). The fitness score is based on the whole genome CRISPR/CAS9 screen (Sidik *et al*., 2016).

**Table S8 – A summary of the results of different attempts to break *T. gondii* tachyzoites using different cell-breaking methods**

| Technique | Brief description | % parasite breakage | | |
| --- | --- | --- | --- | --- |
|  |  | **Experiment 1** | **Experiment 2** | **Experiment 3** |
| Freeze / thaw | Twelve cycles of freezing parasites, in SME-20 buffer (250 mM sucrose, 1mM EDTA, 20 mM MOPS-KOH), into liquid nitrogen for 30 seconds immediately followed by thawing at 37°C for 3 minutes. | 6 cycles - 30%  6 more cycles - 50% | 12 cycles - 50% after |  |
| Freeze / thaw & sonication | 12 cycles of freeze/thaw as above, followed by 2x 5 minutes of sonication at 50/60 Hz with 2 minutes of recovering on ice in between. | 60% |  |  |
| Dounce breaking | 15 strokes of 2 mL dounce on parasites resuspended in SME-20 buffer. | 0 | 0* | 0** |
| Nitrogen cavitation | 2-3 cycles of nitrogen cavitation as detailed in materials and methods. | 1^st^ cycle - 80%  2^nd^ cycle - 99% | 1^st^ cycle - 92%  2^nd^ cycle - 98.7% | 1^st^ cycle – 87.3%  2^nd^ cycle - 98.5% |

* buffer is SME-20/HEPES 1:8 v/v; ** buffer is HEPES.

**Table S9 – Steps taken to generate the 279-gene list**

Separate Excel file
